# Dementia and Risks of Temperature-Related Mortality and Hospitalizations in Germany

**DOI:** 10.1093/gerona/glae292

**Published:** 2024-12-11

**Authors:** Risto Conte Keivabu, Emilio Zagheni, Anne Fink

**Affiliations:** Digital and Computational Demography Lab, Max Planck Institute for Demographic Research, Rostock, Germany; Digital and Computational Demography Lab, Max Planck Institute for Demographic Research, Rostock, Germany; German Center for Neurodegenerative Diseases, Bonn, Germany; (Medical Sciences Section)

**Keywords:** Dementia, Germany, Hospitalization, Mortality, Temperature

## Abstract

**Background:**

Extreme temperatures are associated with negative health outcomes, in particular for older adults with pre-existing conditions. While climate change is expected to increase exposure to temperature levels that are detrimental to health, little is known about how dementia shapes vulnerability to extreme temperatures.

**Methods:**

We leveraged repeated quarterly individual-level health claims from 2004 to 2019 on 250 000 individuals in Germany aged 50 years and above with information on key neurodegenerative diseases such as dementia. We linked data on the location of residence of these individuals with high-resolution gridded meteorological data. In our empirical analysis, we applied an individual-level fixed effects model to estimate how temperature affects the single patient’s probability of hospitalization and death, adjusted for seasonality and comorbidities.

**Results:**

Our findings reveal that heat and cold exposure increases the risk of death. Conversely, the association between extreme temperatures and hospital admissions is more nuanced showing an increase only with cold exposure. Stratifying the analysis by individuals affected by dementia, we observe heat to increase mortality only for individuals with dementia and cold to determine an 8 times larger impact on them and a larger increase in hospitalization. Also, we observe individuals aged above 80 and with dementia to be the most at risk of death with exposure to cold and in particular heat.

**Conclusions:**

Our study contributes to the growing body of evidence on the health impacts of climate change and emphasizes the need for targeted strategies to protect vulnerable groups, particularly patients with dementia, from adverse temperature effects.

Climate-change-associated increases in extreme temperature generate important challenges for public health. A large body of literature has documented an increase in mortality (1–4) and morbidity (5–8) with exposure to high temperatures. In particular, individuals aged 65 and above are the most vulnerable to extreme temperatures, due to their lower thermoregulatory ability and the presence of existing medical conditions that are associated with a decreased capacity to sustain exposure to extreme heat and cold (9–11). Importantly, existing medical conditions such as cardiovascular diseases, diabetes, and respiratory conditions have been found to increase the risk of death and hospitalization with exposure to extreme temperatures for individuals aged above 65 (12,13). However, the role of other conditions, such as dementia syndrome, has been explored less, although it could also increase vulnerability to cold and heat.

The prevalence of dementia has been increasing globally and is currently recognized as the fourth leading cause of death in persons over age 70 (14–16). Dementia is a progressive syndrome caused by several diseases and characterized by cerebral changes that lead to memory loss, cognitive decline, personality changes, and problems in performing daily activities, and some of the top causes of dementia include Alzheimer’s disease, cerebrovascular disease, and Parkinson’s disease (17). Existing research considered how exposure to extreme temperatures affects dementia-related deaths in China (18–20), England (21), Germany (22), and also dementia-related hospitalizations in England (23) and New England (24). Potential mechanisms that explain a higher impact of extreme temperatures on individuals with dementia include impaired thermoregulation, cognitive impairment, medication effects, reduced mobility, communication difficulties, and increased comorbidity (25,26). For example, cognitive deficits can affect people’s ability to recognize and respond appropriately to temperature changes, and they may not remember to drink water, leading to an increased risk of dehydration and heat-related illnesses. Similarly, communication difficulties may lead to delayed recognition and treatment of heat-related conditions (27).

In this article, we leveraged individual-level data on about 250 000 individuals in Germany to analyze how exposure to cold and heat affects hospitalizations and mortality, and how the impact is moderated by dementia. Existing research has shown that cold and heat exposure in Germany is associated with increased mortality (28–30), myocardial infarction (31), and hospital admissions (29,32). These studies highlight the importance of understanding and mitigating the health impacts of temperature extremes in Germany, particularly as climate change is expected to increase the frequency and intensity of such events (33,34). Critically, Germany has a large population of individuals with dementia that is estimated to amount to 1.7 million, expected to increase to 3 million by 2070 (35) and that could be particularly at risk when exposed to extreme temperatures (20).

This study aims to expand our understanding of climate-change-related risks for older adults, particularly examining the impact of extreme temperatures on hospitalization and mortality among individuals with dementia in Germany. Specifically, we investigate 3 main objectives. First, we assess the effects of temperature extremes on mortality among individuals with dementia in Germany from 2004 to 2019, building upon previous research that only covered data up to 2010 (22). Second, we explore temperature-related hospitalization risks and disparities associated with dementia, addressing a gap in the German context where, to our knowledge, no prior studies have examined this relationship. Third, we analyze how dementia moderates the risks of temperature-related mortality and hospitalization, with a focus on heterogeneity across different age groups. Our hypotheses are that individuals with dementia experience higher risks of both mortality and hospitalization during extreme temperature events and that these risks differ by age, with older individuals affected by dementia being the most vulnerable. This study contributes new evidence on how neurodegenerative conditions like dementia influence susceptibility to climate-related health impacts, offering insights that could inform targeted interventions and policies.

## Data and Methods

### Public Health Insurance Data From AOK

We used data from 250 000 insured persons aged 50 and over from Germany’s largest public health insurance fund, the Allgemeine Ortskrankenkassen (AOK). The scientific research institute of the AOK (WIdO) has strict rules regarding data sharing because of the fact that health claims data are a sensitive data source and have ethical restrictions imposed due to concerns regarding privacy. Anonymized data are available to all interested researchers upon request. The 2.2% random sample was drawn at the beginning of 2004 and followed up until the end of 2019. After data cleaning to remove inconsistencies, we had information on 244 186 individuals. The data consist of a demographic section with information on gender, age, region of residence by 5-digit zip code, and, where applicable, date of death. The medical section contains information on all inpatient and outpatient diagnoses coded by ICD-10 and whether a patient was treated in the hospital or not. All information was available on a quarterly basis. Following the assignment of the 5-digit zip code to the districts/municipalities (in German *Kreise*), data on individuals from a total of 398 districts were available. We do not have data for 2 Kreise from the total of 400 due to missing observations in these 2 administrative units.

From these data, we constructed our 2 main outcomes: mortality and hospitalization. Mortality is constructed using the date of death and is binary (1 = Death). For hospitalizations, we constructed a binary variable denoting an individual being hospitalized (1 = Hospitalized) for at least 1 day in a specific quarter.

### Definition of Dementia

We defined dementia as having at least one of the following ICD-10 codes: F00-F03, F05.1, G30, G31.82, and G23.1. The ICD-10 codes referenced include F00-F03 (dementia in Alzheimer’s disease, vascular dementia, and unspecified dementia), F05.1 (delirium superimposed on dementia), G30 (Alzheimer’s disease), G31.82 (Lewy body disease), and G23.1 (progressive supranuclear palsy), and cover various forms of dementia and related neurodegenerative conditions. We used an established internal 2-step validation strategy to overcome the problem of false-positive diagnoses (36,37). First, outpatient diagnoses were marked as verified, and inpatient discharge or secondary diagnoses were identified. Second, diagnoses were considered valid if they were confirmed by a simultaneous inpatient and outpatient diagnosis, by 2 diagnoses from 2 different types of physicians within the same quarter, or by co-occurrence during the study period. A diagnosis was also considered valid if an individual died within the same quarter in which dementia was first diagnosed.

### Covariates From AOK Data

We adjusted our models for the updated version of the weighted Charlson Comorbidity Index (CCI) (38). The index ranges from 0 to 24. Specifically, a score of 0 indicates no comorbid conditions, whereas higher scores correspond to an increasing number and severity of comorbidities. The CCI is widely used to predict the risk of mortality and resource use, with each point increase in the index associated with a higher risk of adverse health outcomes. In analyses with interaction effects with dementia or stratification by dementia status, we used an adapted CCI ranging from 0 to 22 that excludes dementia, as that is already added in the interaction.

### Meteorological Data

We collected data on meteorological variables from the E-OBS database that is freely and openly available in the Copernicus Data Store (CDS). The dataset is gridded with a resolution of about 9 km and provides daily observations on a range of meteorological variables including temperature, precipitation, humidity, solar radiation, and wind speed for the whole European territory. We averaged the values of the gridded meteorological data within each of the 398 Kreise in Germany to compute daily averages.

We captured exposure to temperature for each individual counting the number of days in 11 temperature ranges based on mean daily temperature during each quarter and Kreise. More precisely, the temperature ranges are <−6; −6 to −3; −3 to 0; 0 to 3; 3 to 6; 6 to 9; 9 to 16; 16 to 18; 18 to 21; 21 to 24, and >24°C. The range 9–16°C is our comfort zone and is excluded in our models. We constructed the temperature ranges similarly to previous studies interested in the impact of temperature on mortality or hospitalization (29) that are informed by recent methodological strategies that allow us to capture the nonlinear impacts of temperature on health outcomes (39). The temperature ranges defined in this study are specific to Germany, where extreme temperatures are relatively moderate compared to other regions. In higher-latitude countries like Sweden, lower thresholds would be set for cold temperatures, whereas lower-latitude countries such as Spain would present higher thresholds for hot temperatures. These regional differences are due to acclimatization and variations in temperature–mortality relationships, which shape how populations in different climates respond to temperature extremes.

For the other meteorological variables, which are precipitation, wind speed, humidity, and solar radiation, we averaged the daily values to quarterly values for each Kreise. Precipitation is measured in millimeters (mm), wind speed in meters per second (m/s), humidity in percentages (%), and solar radiation by watts per square meter (W/m^2^).

Finally, we also measured average levels of the air pollutant PM2.5 gathering data provided by the Copernicus Atmosphere Monitoring Service at a resolution of about 75 km. Air pollution is measured as micrograms per cubic meter (µg/m^3^), and we calculated the average monthly values of the gridded data for each of the Kreise and computed quarterly mean values. We introduced air pollution as a control variable due to possible bias in our results when this is not included (40).

### Empirical Strategy

In our analysis, we run a linear probability model with fixed effects described in Equation (1):


$$\begin{array}{l} Y_{ikt}=\;\Sigma \beta TEM{P_{kt}}^{\cdot }+X_{ikt}+\delta_{i}+\upsilon_{q}+\;\mu_{y}+\varepsilon_{ikt} \end{array}$$ (1)

Our outcome *Y* represents the probability of hospitalization/death of individual *i* in Kreise *k* at time *t* (quarter and year). We add a vector *X* of time-varying control variables for CCI at the individual level and contextual controls for average precipitation, wind speed, humidity, solar radiation, and air pollution. To address potential confounding factors, we incorporate a substantial number of fixed effects. Specifically, we include δ as individual-level fixed effects to capture individual-level unobserved heterogeneity, υ as quarter fixed effects to account for seasonal patterns, and μ as year fixed effects to control for year-specific factors. Standard errors are clustered at the location level (Kreise) to account for within-location correlation. Based on such an estimation method, the coefficients can be interpreted as an individual’s change in the probability of hospitalization/death when exposed to an additional day in the described temperature ranges relative to a day in the comfort zone (9–16°C). We acknowledge that OLS models have limitations when analyzing binary outcomes, but compared to a logistic regression it allows a more straightforward interpretation of our results and has some advantages when estimating models with a large number of fixed effects with rare events as outcomes for which logistic models are not always well suited (41,42).

To capture the heterogeneous impact of temperature on our outcomes by dementia, we interacted temperature variables with a binary indicator describing an individual as being diagnosed with dementia (1 = Dementia). We describe such analysis in Equation (2):


$$\begin{array}{l} Y_{ikt}=\;\Sigma \beta TEMP_{kt}\times Dementia_{i}+X_{ikt}+\delta_{i}+{\;\upsilon }_{q}+\mu_{y}+{\;\varepsilon }_{ikt} \end{array}$$ (2)

In this analysis, we proceed similarly to the analysis described in Equation (1), but add an interaction between the temperature bins TEMP and the dementia status of each individual.

The stratified impact of temperature on mortality and hospitalization by dementia might further differ based on age. Age is well documented in the literature to increase the risk of hospitalization and mortality with exposure to temperature extremes (9). Nevertheless, dementia could further increase the risk of mortality and hospitalization determined by age. We constructed a categorical variable for age, respectively, 50–59, 60–69, 70–79, and above 80, and performed the analysis of Equation (2) separately by these age categories.

## Results

In Table 1, we present descriptive statistics of the main variables we used in our analysis based on the total number of 11 001 659 observations from 244 186 individuals followed quarterly from 2004 to 2019. Approximately 1% of our sample experienced death and 9% were hospitalized. The prevalence of dementia in all our observations is 10% and the average age is 71. Considering temperature, as expected, we observe the majority of days to be concentrated in the central temperature range with a lower prevalence at the extremes. Also, in Supplementary Figure 1, we present a map of Germany and the average temperature in each of the Kreise during the period of analysis, 2004–2019. The average level of PM2.5 air pollution is 15 µg/m^3^, well above the WHO limit of 5 µg/m (3,43). The other meteorological variables show an average level of humidity of 78%, solar radiation of 123 W/m^2^, wind speed of 3.2 m/s, and 2.0 (mm) of rain.

**Table 1.** Descriptive Statistics of Mortality, Hospitalizations, Sociodemographic Characteristics, and Environmental Exposures of the AOK Sample in Germany

	Mean (standard deviation)	Range (min–max)	Proportion
Death			0.01
Hospitalization			0.09
Age	71.44 (10.05)	50–110	
CCI with dementia	2.6 (2.67)	0–21	
CCI without dementia	2.39 (2.48)	0–19	
Dementia			0.1
Temperature (*N* days)			
<−6°C	1.28 (3.24)	0–34	
−6 to −3°C	2.57 (4.87)	0–38	
−3 to 0°C	5.76 (8.02)	0–41	
0–3°C	9.52 (10.29)	0–51	
3–6°C	11.21 (10.44)	0–48	
6–9°C	11.9 (9.17)	0–47	
9–16°C	24.42 (14.65)	0–74	
16–18°C	11.98 (12.22)	0–54	
18–21°C	7.97 (9.67)	0–43	
21–24°C	3.61 (5.25)	0–34	
>24°C	1.09 (2.64)	0–27	
Environmental controls			
PM2.5	15.64 (3.97)	5.71–29.94	
Humidity (%)	78.47 (6.62)	58.26–92.53	
Solar radiation(W/m^2^)	123.63 (69.72)	31.34–242.95	
Wind speed (m/s)	3.19 (0.65)	1.15–7.7	
Rain (mm)	2.06 (0.77)	0.3–7.75	
Total Kreise	398		
Total individuals	244 186		
Total observations	11 001 659		

*Notes*: AOK = Allgemeine Ortskrankenkassen; CCI = Charlson Comorbidity Index.

**Figure 1.**
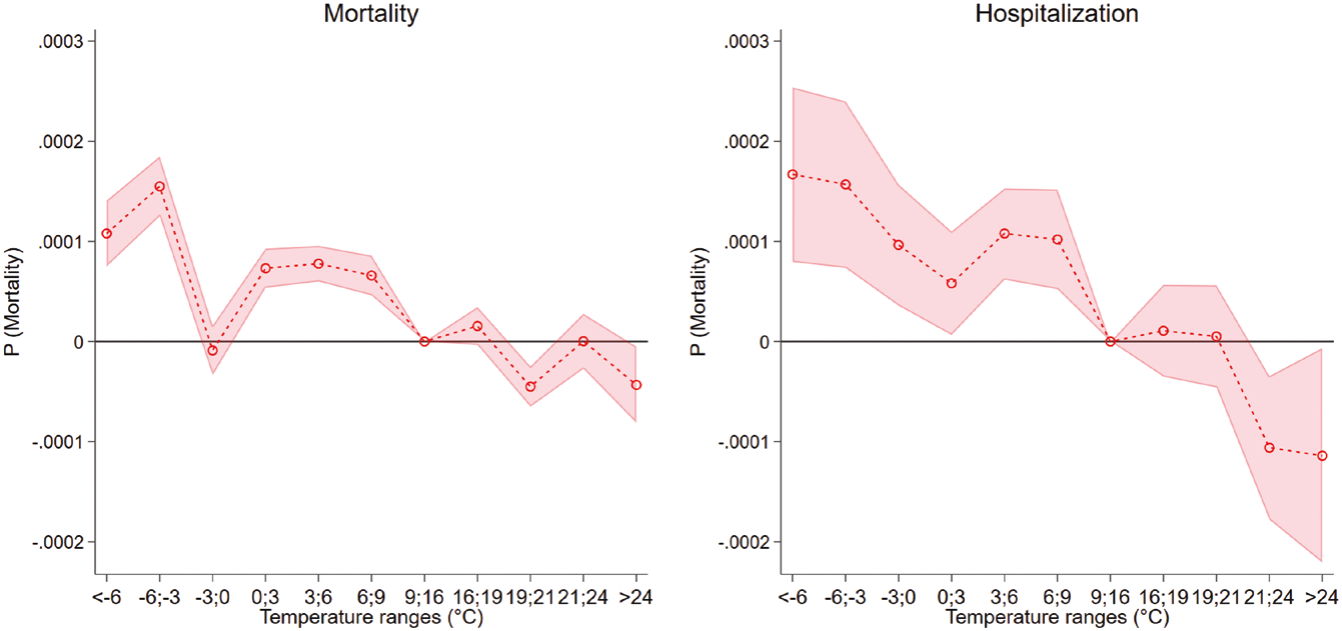
Temperature, hospitalization, and mortality.

In Figure 1 (Supplementary Table 1), we present results based on the model described in Equation (1) for the outcomes of mortality and hospitalization. Here, we observe an increase in the risk of death with exposure to cold days with the highest effect size with the coldest days −6 to −3°C and <−6°C. For instance, an additional day with temperatures from −6 to −3 °C increases mortality by 0.016 and 0.011 percentage points for days <−6°C. Conversely, we do not observe any substantial effects of hot days on mortality, but a small decrease with temperatures >24°C of 0.004 percentage points. Considering hospitalization, we observe a pattern similar to the one observed for mortality. Cold days increase hospitalization and the highest effect size is observed with days <−6°C of 0.017 and 0.016 percentage points with temperatures in the range −6 to −3°C. Hot days do not increase hospitalization, showing to decrease it by −0.011 percentage points with exposure to days 21–24°C or above 24°C. Nevertheless, such findings might mask heterogeneous patterns in the population by dementia.

**Figure 2.**
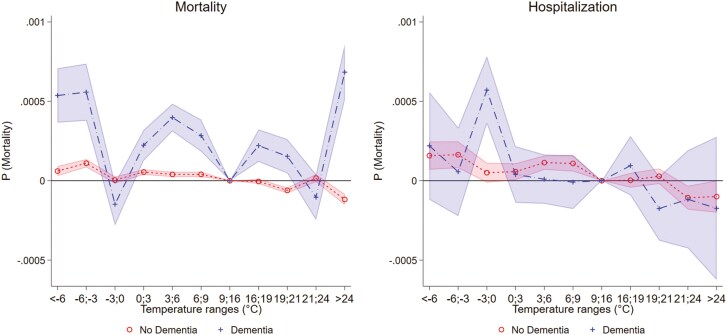
Temperature, hospitalization, and mortality by dementia.

In Figure 2 (Supplementary Table 2), we provide findings on how temperature affects mortality and hospitalization by dementia status. Considering mortality, we observe a stronger susceptibility to exposure to cold and heat for individuals with dementia compared with those without. Individuals with dementia exhibit an increased probability of death by approximately 0.05 percentage points when exposed to an extra day of temperatures below −6°C. In contrast, individuals without dementia show an increase of only 0.006 percentage points, indicating that the effect size for those without dementia is about 8 times smaller. Heat exposure reveals even greater disparities, with individuals with dementia experiencing an increase in the probability of mortality by 0.07 percentage points with days >24°C and larger than other types of temperature exposures. In contrast, those without dementia show a decrease of −0.012 percentage points, suggesting that the effect of heat is concentrated in individuals with dementia. The results for hospitalization look more nuanced. Here, we observe a larger increase in hospitalization with moderate cold exposure (−3 to 0°C) of 0.06 percentage points for individuals with dementia. At lower temperatures, the increase is about 0.016 percentage points with temperature <−6°C for both groups, but with larger statistical uncertainty for individuals with dementia. Considering heat exposure, we do not observe it to substantially increase hospitalizations for both groups.

The impact of cold and hot days on mortality and hospitalization could differ in specific age categories. We first run a model as in Equation (2), but interact with the categorical age variable described in the Methods section with temperature. We report the results of such analysis in Supplementary Table 3 and observe an increase in the risk of mortality and hospitalization with exposure to cold and hot days at higher ages. For instance, individuals aged above 80 show to be the most at risk of mortality and hospitalization when exposed to temperatures <−6°C and with exposure to temperature >24°C.

**Figure 3.**
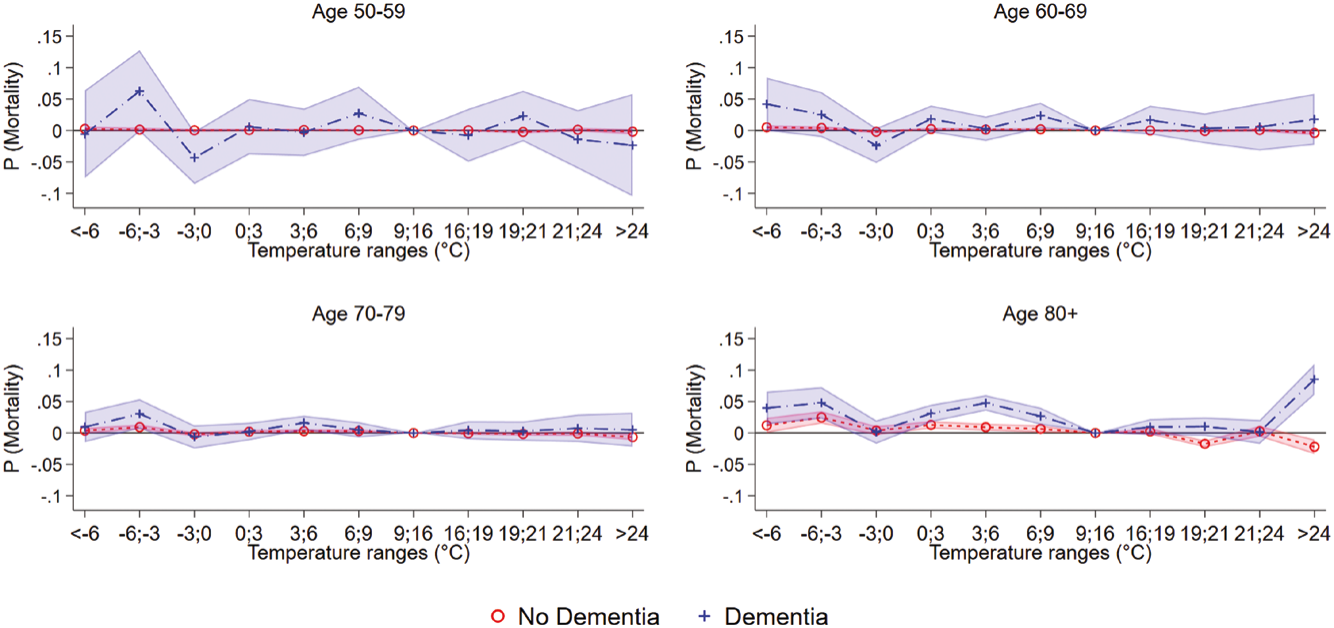
Temperature and mortality by dementia and age.

The stratified impacts of temperature by age might differ by dementia. In Figure 3 (Supplementary Table 4), we report the findings of an analysis based on Equation (2) run separately by the 4 age categories for mortality. Here, we observe a negligible impact of cold and hot days on mortality in the 3 age categories spanning age 50–79, and no differences by dementia. However, for the age category 80+, we observe a higher risk of death with hot days (>24°C) by about 0.09 percentage points for individuals with dementia, but a decrease by −0.02 percentage points for individuals without dementia. Also, we observe a higher risk with moderate cold exposure for individuals with dementia in particular at the temperature range 3 to 0°C.

**Figure 4.**
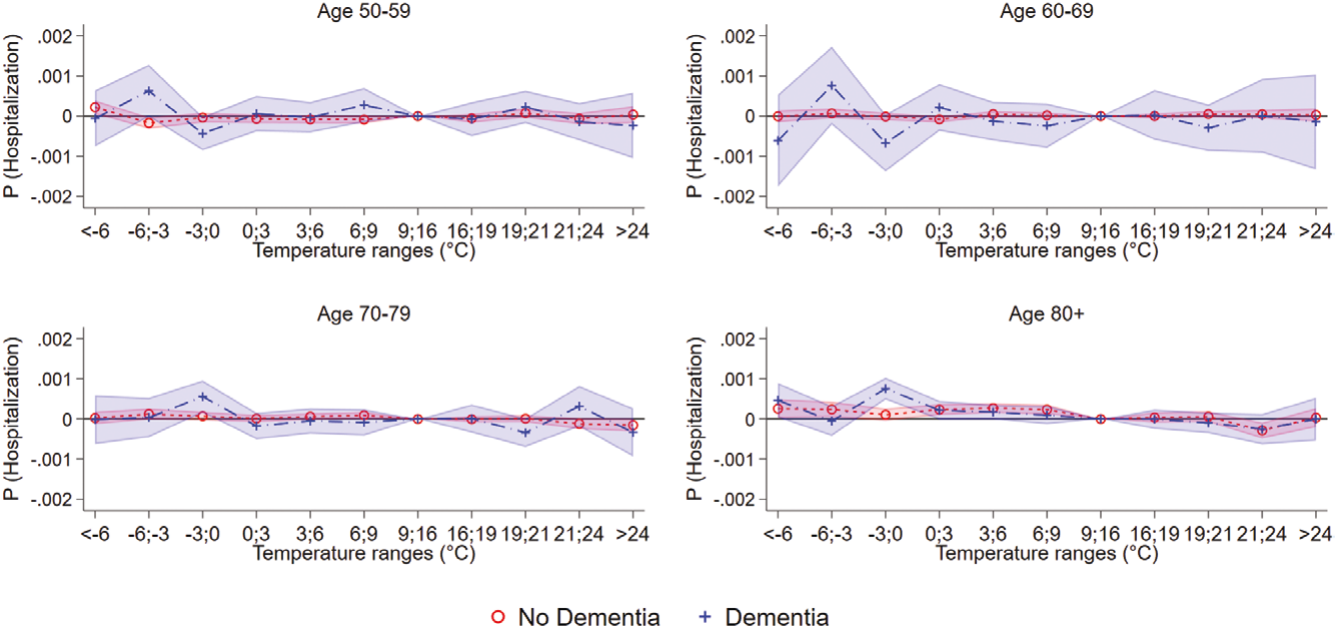
Temperature and hospitalization by dementia and age.

Figure 4 (Supplementary Table 5), we present similar findings of Figure 3 but for hospitalization. Here, we observe the increase in hospitalization to be concentrated in individuals aged 80+ and higher risk for individuals with dementia with temperature −3 to 0°C compared to individuals without dementia.

### Robustness checks

We performed additional analyses to investigate the robustness of the results to alternative modeling choices when estimating how temperature affects individuals’ probability of hospitalization and mortality. For instance, the impacts of cold and heat exposure observed in a specific quarter could determine lagged effects in the subsequent quarters. Existing research has observed a harvesting effect determined by heat and cold exposure, by which increases in death at a specific time of the year due to such weather shocks, would decrease the number of deaths observed later on (22,44,45). Here, we run an analysis consistent with the model in Equation (1), but add the temperature exposure in the previous quarter to our set of variables. In Supplementary Table 6, we observe, for mortality, a decrease in deaths with lagged exposure to moderate cold (−3 to 0; 0 to 3; 3 to 6°C) and with hot days (>24°C) suggesting a harvesting effect of mild cold and hot weather. For hospitalization, we do not observe any major lagged effects. We restricted the definition of hospitalization only to emergency visits as focusing on emergency visits could have the advantage of excluding planned hospital visits such as scheduled operations. In Supplementary Table 7, we observe a similar pattern observed for the broad definition of hospitalizations. Also, we tested the analysis of Equation (1) with an alternative definition of temperature exposure based on a relative measure. A relative measure has the advantage of determining location-specific thresholds for temperature extremes that could better relate to the population adaptation to temperature (46,47). In Supplementary Table 8, we observe similar results, but with lower effect sizes. Finally, we tested alternative fixed effects strategies. More precisely, we ran an analysis with age as a control variable and Kreise-by-month fixed effects. In our main analysis, we did not introduce age as a control variable as we already included year fixed effects in our analysis and further adjusting for age could lead to overfitting. In Supplementary Tables 9 and 10, we added age as a control variable and observed similar results to our main analysis. Also, testing the model with Kreise-by-quartals fixed effects to capture different seasonal patterns based on Kreise, we observed similar results.

## Discussion and Conclusion

Increases in extreme temperature events driven by climate change could pose significant health risks for individuals with dementia, a population projected to grow in the coming years. However, despite their potential vulnerability due to cognitive impairments and underlying health conditions, limited knowledge exists about the risks people with dementia face, which this study addresses by examining the impact of extreme temperatures on mortality and hospitalization in Germany. We obtained 3 main findings. First, we showed evidence of an increase in mortality and hospitalization with exposure to cold days, but mixed results for heat, for which we observed an increase in mortality and a decrease in hospitalizations. Second, we estimated a starker increase in mortality with exposure to cold and heat for individuals with dementia, compared to those without. Nevertheless, we observed the findings for hospitalization to be more nuanced. In fact, we measured an increase in hospitalization with cold days, with this effect being similar for both groups, while moderate cold (−3 to 0°C) was assessed to be more consequential for individuals with dementia. Conversely, heat exposure did not lead to substantially increased hospitalizations for both groups. Third, we provided evidence of the increasing vulnerability to heat and cold by age and observed individuals aged above 80 with dementia to be the most at risk of heat and cold exposure.

Our results align with existing findings in the literature showing an increase in dementia-related deaths with extreme temperatures (18–21), also highlighting the larger vulnerability to heat and cold for individuals with dementia. Interestingly, in the pooled sample of individuals, we did not observe any major impact of hot days on mortality in contrast with other studies (29). Nevertheless, such findings appear to be explained by the age gradient in the impact of extreme temperature: When stratifying the analysis by age categories, we observed mortality to increase only for individuals aged 80+. For hospitalization, 1 study in New England showed an increase in dementia-related hospitalization with cold exposure, but a lack of an effect with hot weather (24). Conversely, a study in England showed an increase in dementia-related hospitalization with hot, but no increase in cold temperature (23). Our results for hospitalization appear to align with those observed in New England, showing an increase in hospitalization only with cold weather. Nevertheless, as we focus on a broad definition of hospitalization, our results are not closely comparable.

Despite a careful study design, this study has limitations that we would like to acknowledge. Due to the fact that approximately 45% of diagnoses are categorized as unspecified dementia (F03), we have not differentiated between types of dementia based on etiology. However, such issues do not determine the misclassification of individuals as having dementia, as shown in previous studies indicating reasonable estimates of dementia prevalence and incidence based on the AOK data (36,48). The AOK data are not fully representative of the German population of individuals aged above 50. In fact, it has a higher proportion of individuals with low income and low educational attainment compared to private or other public health insurers in Germany, which may reflect a higher prevalence of morbidity (49). However, health claims data are only marginally biased by attrition for reasons other than death because the data are complete over time and the rate of change between public health insurance funds is low, especially at the oldest ages. The medical diagnoses were not subject to recall bias. Selection bias by healthcare providers or self-selection into the study can be ruled out. In addition, all insured persons were included in the study, regardless of their functional and cognitive status. We were unable to test the mechanisms that might determine a higher risk of death and hospitalizations for individuals with dementia due to the lack of relevant data. Similarly, we recognize that additional factors influencing the relationships between dementia, hospitalization, and mortality—such as income, nutrition, frailty, access to resources, and stable housing—are not addressed in our analysis. These variables could significantly alter or mitigate the risks we have observed. For example, beyond education and income, housing conditions play a vital role; individuals with dementia who live alone face a higher risk of dehydration during hot weather due to the lack of support. However, our dataset does not include these variables, which constrains our ability to fully assess their impact.

Further studies could improve upon our work in three main ways. First, our study is representative of the German individuals insured by AOK, the largest insurer in Germany. Future studies could leverage similar data on other countries and climatic contexts to analyze how extreme temperature affects mortality and hospitalization and how this differs by dementia. Also, additional information on the individual housing or socioeconomic status could provide a deeper insight into the factors explaining our results. Second, we ran the analysis based on an unspecified classification of dementia, but further studies could inquire how specific types of dementia affect the risk of hospitalization and mortality with exposure to extreme temperatures. Possibly, such analysis could also shed light on the mechanisms that determine a higher risk of mortality and hospitalization for individuals with dementia. Third, in this study, we inquired how dementia increases the risks of hospitalization and death determined by extreme temperature, but temperature could be a contributing factor in the onset of dementia (50).

Our findings highlight critical public health challenges resulting from the combination of rising extreme temperatures due to climate change and the growing prevalence of dementia. This convergence requires the need for targeted interventions to support vulnerable populations like those with dementia, who face amplified risks during heat waves due to physiological sensitivities and cognitive impairments that can hinder their ability to recognize and respond to dangerous temperatures. Additionally, while extreme cold days are decreasing, the number of people with dementia affected by these events is increasing, warranting similar attention to cold weather risks.

In conclusion, addressing these risks requires the development of specific public health and clinical measures. Public health policies should prioritize expanding access to cooling centers during heat waves and warming centers during cold snaps, alongside specialized alert systems that notify caregivers of upcoming extreme weather events. Clinically, healthcare providers should receive training to identify and manage both heat- and cold-related symptoms in dementia patients, preparing for increased service demands during extreme temperature events. By integrating dementia care into broader climate resilience strategies, we can enhance the safety and well-being of this population throughout various extreme weather conditions.

## Supplementary Material

glae292_suppl_Supplementary_Material

## Data Availability

Interested individuals or an institution who wish to request access to the health claims data of the AOK may contact the WIdO (webpage: http://www.wido.de/, mail: wido@wido.bv.aok.de). The meteorological data are accessible in the Copernicus Data Store at the link: https://cds.climate.copernicus.eu/#!/home.
