# Supplementary material for: Dementia and Risks of Temperature-Related Mortality and Hospitalizations in Germany
Source: J Gerontol A Biol Sci Med Sci. 2024 Dec 11;80(4):glae292. doi: 10.1093/gerona/glae292 (PMC11949426; doi:10.1093/gerona/glae292)
Supplement: glae292_suppl_Supplementary_Material [file glae292_suppl_supplementary_material.docx]

**Supplementary materials:** **Dementia and risks of temperature-related mortality and hospitalizations in Germany**

Table of contents:

- Tables: S1-S10;
- Figures: Figure S1.

Table S1. Temperature, mortality and hospitalizations

|  | Mortality |  |  |  |  | Hospitalization |  |  |  |  |  |
| --- | --- | --- | --- | --- | --- | --- | --- | --- | --- | --- | --- |
| Variables | Coeff. | Standard Error | T-Stat. | P-value | Sign. | Coeff. | Standard Error | T-Stat. | P-value | Sign. |  |
|  |  |  |  |  |  |  |  |  |  |  |  |
| < -6 °C | 0.00011 | (0.00002) | 6.41361 | 0.00000 | *** | 0.00017 | (0.00004) | 3.75217 | 0.00020 | *** |  |
| -6 °C to -3 °C | 0.00016 | (0.00002) | 10.26366 | 0.00000 | *** | 0.00016 | (0.00004) | 3.69814 | 0.00025 | *** |  |
| -3 °C to 0 °C | -0.00001 | (0.00001) | -0.69639 | 0.48659 |  | 0.00010 | (0.00003) | 3.12253 | 0.00192 | *** |  |
| 0 °C to 3 °C | 0.00007 | (0.00001) | 7.32869 | 0.00000 | *** | 0.00006 | (0.00003) | 2.20877 | 0.02776 | ** |  |
| 3 °C to 6 °C | 0.00008 | (0.00001) | 8.54607 | 0.00000 | *** | 0.00011 | (0.00002) | 4.61075 | 0.00001 | *** |  |
| 6 °C to 9 °C | 0.00007 | (0.00001) | 6.50009 | 0.00000 | *** | 0.00010 | (0.00003) | 4.01959 | 0.00007 | *** |  |
| 16 °C to 18 °C | 0.00002 | (0.00001) | 1.60055 | 0.11027 |  | 0.00001 | (0.00002) | 0.46136 | 0.64479 |  |  |
| 18 °C to 21 °C | -0.00005 | (0.00001) | -4.36545 | 0.00002 | *** | 0.00001 | (0.00003) | 0.19680 | 0.84409 |  |  |
| 21 °C to 24 °C | 0.00000 | (0.00001) | 0.02195 | 0.98250 |  | -0.00011 | (0.00004) | -2.90496 | 0.00388 | *** |  |
| > 24 °C | -0.00004 | (0.00002) | -2.22358 | 0.02674 | ** | -0.00011 | (0.00005) | -2.08280 | 0.03791 | ** |  |
| CCI | 0.01196 | (0.00011) | 109.23972 | 0.00000 |  | 0.03408 | (0.00024) | 139.39563 | 0.00000 | *** |  |
| Wind | 0.00004 | (0.00019) | 0.23121 | 0.81727 |  | 0.00013 | (0.00049) | 0.25680 | 0.79746 |  |  |
| Rain | 0.00005 | (0.00007) | 0.74096 | 0.45915 |  | 0.00056 | (0.00018) | 3.17305 | 0.00163 | *** |  |
| Solar radiation | 0.00002 | (0.00001) | 3.16072 | 0.00169 | *** | 0.00001 | (0.00002) | 0.77843 | 0.43677 |  |  |
| Relative Humidity | -0.00002 | (0.00002) | -0.66787 | 0.50460 |  | -0.00014 | (0.00007) | -2.02586 | 0.04344 | ** |  |
| PM2.5 | 0.00012 | (0.00003) | 3.84724 | 0.00014 | *** | -0.00006 | (0.00011) | -0.50154 | 0.61627 |  |  |
| Constant | -0.02671 | (0.00253) | -10.53897 | 0.00000 | *** | 0.00656 | (0.00682) | 0.96220 | 0.33653 |  |  |
|  |  |  |  |  |  |  |  |  |  |  |  |
| Observations | 11,001,659 |  |  |  |  | 11,001,659 |  |  |  |  |  |
| R^2^ | 0.08525 |  |  |  |  | 0.13571 |  |  |  |  |  |
| Adjusted R^2^ | 0.0645 |  |  |  |  | 0.116 |  |  |  |  |  |
| F-stat | 992.3 |  |  |  |  | 1412 |  |  |  |  |  |
| F-test | 0 |  |  |  |  | 0 |  |  |  |  |  |

Note: we present results for running an analysis as in Equation (1). Standard errors clustered at the Kreise level. Abbreviations: CCI: Charlson Comorbidity Index; Coeff: coefficient, F-Stat.: F-Statistics; Sign.: Significance. *** p<0.01, ** p<0.05, * p<0.1.

Table S2. Temperature, mortality, hospitalizations and interaction with Dementia

|  | Mortality |  |  |  |  | Hospitalizations |  |  |  |  |
| --- | --- | --- | --- | --- | --- | --- | --- | --- | --- | --- |
| Variables | Coeff. | Standard Error | T-Stat. | P-value | Sign. | Coeff. | Standard Error | T-Stat. | P-value | Sign. |
| < -6 °C | 0.00006 | (0.00002) | 3.82719 | 0.00015 | *** | 0.00016 | (0.00005) | 3.50330 | 0.00051 | *** |
| Dementia | 0.02716 | (0.00167) | 16.24904 | 0.00000 | *** | 0.07182 | (0.00326) | 22.04059 | 0.00000 | *** |
| Dementia#< -6 °C | 0.00048 | (0.00009) | 5.45299 | 0.00000 | *** | 0.00006 | (0.00018) | 0.34857 | 0.72759 |  |
| -6 °C to -3 °C | 0.00011 | (0.00001) | 8.35533 | 0.00000 | *** | 0.00016 | (0.00004) | 3.81676 | 0.00016 | *** |
| Dementia#-6 °C to -3 °C | 0.00045 | (0.00009) | 4.92284 | 0.00000 | *** | -0.00011 | (0.00014) | -0.75005 | 0.45367 |  |
| -3 °C to 0 °C | 0.00000 | (0.00001) | 0.40804 | 0.68347 |  | 0.00005 | (0.00003) | 1.59549 | 0.11139 |  |
| Dementia#-3 °C to 0 °C | -0.00015 | (0.00007) | -2.26786 | 0.02387 | ** | 0.00052 | (0.00011) | 4.66375 | 0.00000 | *** |
| 0 °C to 3 °C | 0.00005 | (0.00001) | 6.13858 | 0.00000 | *** | 0.00006 | (0.00003) | 2.13402 | 0.03345 | ** |
| Dementia#0 °C to 3 °C | 0.00017 | (0.00005) | 3.45479 | 0.00061 | *** | -0.00002 | (0.00009) | -0.19374 | 0.84648 |  |
| 3 °C to 6 °C | 0.00004 | (0.00001) | 4.88067 | 0.00000 | *** | 0.00011 | (0.00002) | 5.02479 | 0.00000 | *** |
| Dementia#3 °C to 6 °C | 0.00036 | (0.00004) | 8.16548 | 0.00000 | *** | -0.00010 | (0.00008) | -1.34292 | 0.18006 |  |
| 6 °C to 9 °C | 0.00004 | (0.00001) | 4.64402 | 0.00000 | *** | 0.00011 | (0.00003) | 4.16708 | 0.00004 | *** |
| Dementia#6 °C to 9 °C | 0.00024 | (0.00005) | 4.87653 | 0.00000 | *** | -0.00012 | (0.00009) | -1.32778 | 0.18501 |  |
| 16 °C to 18 °C | -0.00000 | (0.00001) | -0.58808 | 0.55681 |  | 0.00000 | (0.00002) | 0.12023 | 0.90436 |  |
| Dementia#16 °C to 18 °C | 0.00023 | (0.00005) | 4.42465 | 0.00001 | *** | 0.00009 | (0.00010) | 0.94404 | 0.34572 |  |
| 18 °C to 21 °C | -0.00006 | (0.00001) | -6.62753 | 0.00000 | *** | 0.00003 | (0.00002) | 1.14306 | 0.25370 |  |
| Dementia#18 °C to 21 °C | 0.00021 | (0.00005) | 3.92702 | 0.00010 | *** | -0.00020 | (0.00010) | -2.01527 | 0.04455 | ** |
| 21 °C to 24 °C | 0.00002 | (0.00001) | 1.26263 | 0.20746 |  | -0.00011 | (0.00004) | -2.80127 | 0.00534 | *** |
| Dementia#21 °C to 24 °C | -0.00012 | (0.00007) | -1.62725 | 0.10447 |  | -0.00001 | (0.00016) | -0.06040 | 0.95187 |  |
| > 24 °C | -0.00012 | (0.00002) | -6.68045 | 0.00000 | *** | -0.00010 | (0.00005) | -1.94461 | 0.05252 | * |
| Dementia#> 24 °C | 0.00080 | (0.00009) | 8.93500 | 0.00000 | *** | -0.00007 | (0.00023) | -0.32533 | 0.74510 |  |
| CCI without dementia | 0.01078 | (0.00012) | 90.50533 | 0.00000 | *** | 0.03413 | (0.00027) | 127.20988 | 0.00000 | *** |
| Wind | -0.00000 | (0.00018) | -0.00824 | 0.99343 |  | 0.00012 | (0.00049) | 0.24539 | 0.80628 |  |
| Rain | 0.00004 | (0.00007) | 0.56786 | 0.57045 |  | 0.00056 | (0.00018) | 3.15870 | 0.00171 | *** |
| Solar radiation | 0.00002 | (0.00001) | 3.11572 | 0.00197 | *** | 0.00001 | (0.00002) | 0.71942 | 0.47230 |  |
| Relative Humidity | -0.00000 | (0.00002) | -0.20915 | 0.83444 |  | -0.00014 | (0.00007) | -2.09248 | 0.03703 | ** |
| PM2.5 | 0.00010 | (0.00003) | 3.38507 | 0.00078 | *** | -0.00006 | (0.00011) | -0.53275 | 0.59450 |  |
| Constant | -0.02420 | (0.00247) | -9.79518 | 0.00000 | *** | 0.00751 | (0.00681) | 1.10354 | 0.27046 |  |
|  |  |  |  |  |  |  |  |  |  |  |
| Observations | 11,001,659 |  |  |  |  | 11,001,659 |  |  |  |  |
| R-squared | 0.08573 |  |  |  |  | 0.13553 |  |  |  |  |
| Adj. R-Squared. | 0.0650 |  |  |  |  | 0.116 |  |  |  |  |
| F-stat | 1041 |  |  |  |  | 921.1 |  |  |  |  |
| F-test | 0 |  |  |  |  | 0 |  |  |  |  |

Note: we present results for running an analysis as in Equation (2). Standard errors clustered at the Kreise level. Abbreviations: CCI: Charlson Comorbidity Index; Coeff: coefficient, F-Stat.: F-Statistics; Sign.: Significance. *** p<0.01, ** p<0.05, * p<0.1.

Table S3. Temperature, mortality, hospitalizations and interaction with age

|  | Mortality |  |  |  |  | Hospitalization |  |  |  |  |
| --- | --- | --- | --- | --- | --- | --- | --- | --- | --- | --- |
| Variables | Coeff. | Standard Error | T-Stat. | P-value | Sign. | Coeff. | Standard Error | T-Stat. | P-value | Sign. |
| < -6 °C | -0.00023 | (0.00003) | -8.30907 | 0.00000 | *** | 0.00021 | (0.00008) | 2.70409 | 0.00714 | *** |
| Age 60-69 | -0.01706 | (0.00040) | -42.94506 | 0.00000 | *** | -0.01134 | (0.00181) | -6.27619 | 0.00000 | *** |
| Age 70-79 | -0.03290 | (0.00057) | -57.91857 | 0.00000 | *** | -0.01438 | (0.00206) | -6.97407 | 0.00000 | *** |
| Age 80+ | -0.04882 | (0.00099) | -49.25691 | 0.00000 | *** | -0.02032 | (0.00253) | -8.02196 | 0.00000 | *** |
| Age 60-69 #< -6 °C | 0.00025 | (0.00002) | 9.91509 | 0.00000 | *** | -0.00007 | (0.00010) | -0.75235 | 0.45229 |  |
| Age 70-79 #< -6 °C | 0.00033 | (0.00003) | 10.39316 | 0.00000 | *** | -0.00011 | (0.00010) | -1.15498 | 0.24879 |  |
| Age 80+ #< -6 °C | 0.00070 | (0.00006) | 12.08598 | 0.00000 | *** | 0.00006 | (0.00012) | 0.54141 | 0.58853 |  |
| -6 °C to -3 °C | -0.00018 | (0.00002) | -8.82958 | 0.00000 | *** | -0.00007 | (0.00007) | -1.10123 | 0.27146 |  |
| Age 60-69 #-6 °C to -3 °C | 0.00018 | (0.00002) | 9.03708 | 0.00000 | *** | 0.00027 | (0.00008) | 3.52023 | 0.00048 | *** |
| Age 70-79 #-6 °C to -3 °C | 0.00035 | (0.00003) | 12.58184 | 0.00000 | *** | 0.00025 | (0.00008) | 3.04011 | 0.00252 | *** |
| Age 80+ #-6 °C to -3 °C | 0.00076 | (0.00007) | 10.97216 | 0.00000 | *** | 0.00025 | (0.00010) | 2.43137 | 0.01548 | ** |
| -3 °C to 0 °C | 0.00011 | (0.00002) | 6.23696 | 0.00000 | *** | 0.00006 | (0.00005) | 1.16361 | 0.24528 |  |
| Age 60-69 #-3 °C to 0 °C | -0.00004 | (0.00002) | -2.42913 | 0.01558 | ** | 0.00000 | (0.00006) | 0.01235 | 0.99016 |  |
| Age 70-79 #-3 °C to 0 °C | -0.00009 | (0.00002) | -4.01452 | 0.00007 | *** | 0.00004 | (0.00006) | 0.67169 | 0.50217 |  |
| Age 80+ #-3 °C to 0 °C | -0.00038 | (0.00005) | -7.92904 | 0.00000 | *** | 0.00007 | (0.00008) | 0.90157 | 0.36783 |  |
| 0 °C to 3 °C | 0.00003 | (0.00002) | 1.94506 | 0.05247 | * | 0.00007 | (0.00005) | 1.51240 | 0.13122 |  |
| Age 60-69 #0 °C to 3 °C | 0.00001 | (0.00001) | 0.61496 | 0.53893 |  | -0.00004 | (0.00005) | -0.86778 | 0.38604 |  |
| Age 70-79 #0 °C to 3 °C | 0.00000 | (0.00002) | 0.24710 | 0.80496 |  | -0.00004 | (0.00006) | -0.74316 | 0.45782 |  |
| Age 80+ #0 °C to 3 °C | 0.00018 | (0.00004) | 4.97185 | 0.00000 | *** | 0.00006 | (0.00007) | 0.96950 | 0.33288 |  |
| 3 °C to 6 °C | -0.00014 | (0.00001) | -11.50594 | 0.00000 | *** | -0.00007 | (0.00004) | -1.68756 | 0.09228 | * |
| Age 60-69 #3 °C to 6 °C | 0.00010 | (0.00001) | 8.84177 | 0.00000 | *** | 0.00017 | (0.00005) | 3.34616 | 0.00090 | *** |
| Age 70-79 #3 °C to 6 °C | 0.00020 | (0.00002) | 12.64496 | 0.00000 | *** | 0.00020 | (0.00005) | 3.82219 | 0.00015 | *** |
| Age 80+ #3 °C to 6 °C | 0.00053 | (0.00003) | 17.82000 | 0.00000 | *** | 0.00026 | (0.00006) | 4.53613 | 0.00001 | *** |
| 6 °C to 9 °C | -0.00015 | (0.00001) | -10.66302 | 0.00000 | *** | -0.00007 | (0.00004) | -1.60007 | 0.11038 |  |
| Age 60-69 #6 °C to 9 °C | 0.00013 | (0.00001) | 10.96227 | 0.00000 | *** | 0.00015 | (0.00005) | 2.97639 | 0.00309 | *** |
| Age 70-79 #6 °C to 9 °C | 0.00022 | (0.00002) | 12.99097 | 0.00000 | *** | 0.00020 | (0.00005) | 3.81135 | 0.00016 | *** |
| Age 80+ #6 °C to 9 °C | 0.00048 | (0.00003) | 15.03398 | 0.00000 | *** | 0.00024 | (0.00006) | 3.98609 | 0.00008 | *** |
| 16 °C to 18 °C | -0.00023 | (0.00002) | -12.13546 | 0.00000 | *** | -0.00021 | (0.00005) | -4.58182 | 0.00001 | *** |
| Age 60-69 #16 °C to 18 °C | 0.00017 | (0.00002) | 10.47415 | 0.00000 | *** | 0.00018 | (0.00005) | 3.40704 | 0.00072 | *** |
| Age 70-79 #16 °C to 18 °C | 0.00026 | (0.00002) | 11.17662 | 0.00000 | *** | 0.00024 | (0.00006) | 3.97381 | 0.00008 | *** |
| Age 80+ #16 °C to 18 °C | 0.00046 | (0.00004) | 12.53752 | 0.00000 | *** | 0.00036 | (0.00007) | 4.87377 | 0.00000 | *** |
| 18 °C to 21 °C | -0.00021 | (0.00002) | -12.96276 | 0.00000 | *** | -0.00008 | (0.00005) | -1.53658 | 0.12519 |  |
| Age 60-69 #18 °C to 21 °C | 0.00009 | (0.00002) | 5.36483 | 0.00000 | *** | 0.00004 | (0.00006) | 0.71336 | 0.47604 |  |
| Age 70-79 #18 °C to 21 °C | 0.00017 | (0.00002) | 8.35638 | 0.00000 | *** | 0.00009 | (0.00006) | 1.42529 | 0.15486 |  |
| Age 80+ #18 °C to 21 °C | 0.00039 | (0.00003) | 11.44002 | 0.00000 | *** | 0.00019 | (0.00007) | 2.75533 | 0.00613 | *** |
| 21 °C to 24 °C | 0.00022 | (0.00002) | 9.01055 | 0.00000 | *** | 0.00004 | (0.00007) | 0.52419 | 0.60044 |  |
| Age 60-69 #21 °C to 24 °C | -0.00016 | (0.00002) | -8.11181 | 0.00000 | *** | -0.00007 | (0.00008) | -0.89084 | 0.37355 |  |
| Age 70-79 #21 °C to 24 °C | -0.00025 | (0.00003) | -9.32305 | 0.00000 | *** | -0.00010 | (0.00009) | -1.10900 | 0.26810 |  |
| Age 80+ #21 °C to 24 °C | -0.00031 | (0.00005) | -5.78437 | 0.00000 | *** | -0.00035 | (0.00011) | -3.24854 | 0.00126 | *** |
| > 24 °C | -0.00029 | (0.00004) | -7.13155 | 0.00000 | *** | -0.00017 | (0.00010) | -1.71936 | 0.08632 | * |
| Age 60-69 #> 24 °C | 0.00001 | (0.00003) | 0.40160 | 0.68820 |  | -0.00003 | (0.00011) | -0.26513 | 0.79104 |  |
| Age 70-79 #> 24 °C | 0.00015 | (0.00004) | 4.17668 | 0.00004 | *** | 0.00002 | (0.00011) | 0.18724 | 0.85157 |  |
| Age 80+ #> 24 °C | 0.00090 | (0.00007) | 12.05430 | 0.00000 | *** | 0.00033 | (0.00014) | 2.43030 | 0.01553 | ** |
| CCI | 0.01166 | (0.00011) | 104.93361 | 0.00000 | *** | 0.03397 | (0.00025) | 136.36111 | 0.00000 | *** |
| Wind | 0.00006 | (0.00019) | 0.31459 | 0.75324 |  | 0.00014 | (0.00049) | 0.28263 | 0.77761 |  |
| Rain | 0.00002 | (0.00007) | 0.26267 | 0.79294 |  | 0.00052 | (0.00018) | 2.96080 | 0.00325 | *** |
| Solar radiation | 0.00002 | (0.00001) | 2.94843 | 0.00338 | *** | 0.00001 | (0.00002) | 0.60554 | 0.54517 |  |
| Relative Humidity | 0.00001 | (0.00002) | 0.30204 | 0.76278 |  | -0.00013 | (0.00007) | -1.97791 | 0.04863 | ** |
| PM2.5 | 0.00009 | (0.00003) | 2.98822 | 0.00298 | *** | -0.00007 | (0.00011) | -0.65763 | 0.51115 |  |
| Constant | 0.00013 | (0.00251) | 0.05227 | 0.95834 |  | 0.02016 | (0.00706) | 2.85470 | 0.00453 | *** |
|  |  |  |  |  |  |  |  |  |  |  |
| Observations | 11,001,659 |  |  |  |  | 11,001,659 |  |  |  |  |
| R-squared | 0.08655 |  |  |  |  | 0.13573 |  |  |  |  |
| Adj. R-Squared. | 0.0658 |  |  |  |  | 0.116 |  |  |  |  |
| F-stat | 937.1 |  |  |  |  | 639.5 |  |  |  |  |
| F-test | 0 |  |  |  |  | 0 |  |  |  |  |

Note: we present results for running an analysis as in Equation (2), but with an interaction with age categories:50-59;60-69;70-79;80+. Standard errors clustered at the Kreise level. Abbreviations: CCI: Charlson Comorbidity Index; Coeff: coefficient, F-Stat.: F-Statistics; Sign.: Significance. *** p<0.01, ** p<0.05, * p<0.1.

Table S4. Temperature, mortality separately by age and interaction with dementia

|  | Age 50-59 |  |  |  |  | Age 60-69 |  |  |  |  | Age 70-79 |  |  |  |  | Age 80+ |  |  |  |  |
| --- | --- | --- | --- | --- | --- | --- | --- | --- | --- | --- | --- | --- | --- | --- | --- | --- | --- | --- | --- | --- |
| Variables | Coeff. | Stand. Error | T-Stat. | P-value | Sign. | Coeff. | Stand. Error | T-Stat. | P-value | Sign. | Coeff. | Stand. Error | T-Stat. | P-value | Sign. | Coeff. | Stand. Error | T-Stat. | P-value | Sign. |
|  |  |  |  |  |  |  |  |  |  |  |  |  |  |  |  |  |  |  |  |  |
| < -6 °C | 0.00003 | (0.00002) | 1.34771 | 0.17852 |  | 0.00005 | (0.00002) | 2.93963 | 0.00348 | *** | 0.00004 | (0.00002) | 1.63868 | 0.10207 |  | 0.00012 | (0.00006) | 1.99224 | 0.04703 | ** |
| Dementia | 0.00606 | (0.00692) | 0.87661 | 0.38123 |  | 0.01254 | (0.00362) | 3.46436 | 0.00059 | *** | 0.01866 | (0.00209) | 8.93444 | 0.00000 | *** | 0.02639 | (0.00245) | 10.77920 | 0.00000 | *** |
| Dementia# < -6 °C | -0.00008 | (0.00035) | -0.23725 | 0.81259 |  | 0.00037 | (0.00022) | 1.71175 | 0.08772 | * | 0.00006 | (0.00013) | 0.45677 | 0.64809 |  | 0.00027 | (0.00014) | 1.95824 | 0.05090 | * |
| -6 °C to -3 °C | 0.00001 | (0.00002) | 0.91931 | 0.35849 |  | 0.00004 | (0.00001) | 2.87639 | 0.00424 | *** | 0.00009 | (0.00002) | 4.56798 | 0.00001 | *** | 0.00025 | (0.00005) | 4.88411 | 0.00000 | *** |
| Dementia# -6 °C to -3 °C | 0.00061 | (0.00033) | 1.85765 | 0.06396 | * | 0.00021 | (0.00018) | 1.15885 | 0.24721 | * | 0.00021 | (0.00012) | 1.73688 | 0.08318 | * | 0.00023 | (0.00013) | 1.76701 | 0.07799 | * |
| -3 °C to 0 °C | 0.00000 | (0.00001) | 0.26997 | 0.78732 |  | -0.00002 | (0.00001) | -1.72239 | 0.08577 |  | -0.00002 | (0.00002) | -1.20396 | 0.22932 |  | 0.00004 | (0.00004) | 1.01791 | 0.30934 |  |
| Dementia#-3 °C to 0 °C | -0.00044 | (0.00021) | -2.04933 | 0.04108 | ** | -0.00022 | (0.00014) | -1.51632 | 0.13023 |  | -0.00004 | (0.00010) | -0.45268 | 0.65103 |  | -0.00002 | (0.00010) | -0.23510 | 0.81425 |  |
| 0 °C to 3 °C | 0.00001 | (0.00001) | 0.62840 | 0.53010 |  | 0.00002 | (0.00001) | 2.39060 | 0.01728 | ** | 0.00002 | (0.00001) | 1.48312 | 0.13883 |  | 0.00013 | (0.00003) | 4.22872 | 0.00003 | *** |
| Dementia#0 °C to 3 °C | 0.00005 | (0.00022) | 0.23895 | 0.81127 |  | 0.00016 | (0.00011) | 1.45646 | 0.14605 |  | 0.00000 | (0.00007) | 0.04615 | 0.96322 |  | 0.00018 | (0.00007) | 2.60732 | 0.00947 | *** |
| 3 °C to 6 °C | 0.00001 | (0.00001) | 0.77138 | 0.44094 |  | 0.00001 | (0.00001) | 1.51631 | 0.13023 |  | 0.00003 | (0.00001) | 2.16807 | 0.03074 | ** | 0.00009 | (0.00003) | 3.17262 | 0.00163 | *** |
| Dementia#3 °C to 6 °C | -0.00004 | (0.00019) | -0.19204 | 0.84781 |  | 0.00001 | (0.00010) | 0.13955 | 0.88908 |  | 0.00013 | (0.00006) | 2.22008 | 0.02697 | ** | 0.00039 | (0.00006) | 5.92645 | 0.00000 | *** |
| 6 °C to 9 °C | 0.00000 | (0.00001) | 0.41254 | 0.68016 |  | 0.00002 | (0.00001) | 2.13178 | 0.03364 | ** | 0.00003 | (0.00001) | 2.27089 | 0.02369 | ** | 0.00007 | (0.00003) | 2.22484 | 0.02665 | ** |
| Dementia#6 °C to 9 °C | 0.00027 | (0.00022) | 1.24515 | 0.21381 |  | 0.00022 | (0.00011) | 2.05631 | 0.04040 | ** | 0.00002 | (0.00006) | 0.32339 | 0.74657 |  | 0.00020 | (0.00007) | 2.83453 | 0.00482 | *** |
| 16 °C to 18 °C | 0.00000 | (0.00001) | 0.31180 | 0.75535 |  | -0.00000 | (0.00001) | -0.08753 | 0.93029 |  | -0.00001 | (0.00001) | -0.41523 | 0.67820 |  | 0.00003 | (0.00003) | 0.98937 | 0.32308 |  |
| Dementia#16 °C to 18 °C | -0.00008 | (0.00021) | -0.37412 | 0.70852 |  | 0.00017 | (0.00012) | 1.42924 | 0.15372 |  | 0.00005 | (0.00008) | 0.65504 | 0.51282 |  | 0.00007 | (0.00007) | 1.04099 | 0.29851 |  |
| 18 °C to 21 °C | -0.00002 | (0.00001) | -2.33301 | 0.02014 | ** | -0.00001 | (0.00001) | -0.78684 | 0.43184 |  | -0.00002 | (0.00001) | -1.11332 | 0.26624 |  | -0.00017 | (0.00003) | -6.03472 | 0.00000 | *** |
| Dementia#18 °C to 21 °C | 0.00025 | (0.00021) | 1.23153 | 0.21885 |  | 0.00004 | (0.00012) | 0.33677 | 0.73647 |  | 0.00005 | (0.00008) | 0.56669 | 0.57125 |  | 0.00027 | (0.00008) | 3.61424 | 0.00034 | *** |
| 21 °C to 24 °C | 0.00001 | (0.00001) | 0.85891 | 0.39091 |  | 0.00001 | (0.00001) | 0.58794 | 0.55690 |  | -0.00001 | (0.00002) | -0.52848 | 0.59746 |  | 0.00003 | (0.00004) | 0.59985 | 0.54895 |  |
| Dementia#21 °C to 24 °C | -0.00015 | (0.00024) | -0.64835 | 0.51713 |  | 0.00005 | (0.00019) | 0.25369 | 0.79987 |  | 0.00008 | (0.00011) | 0.74753 | 0.45518 |  | -0.00001 | (0.00010) | -0.09302 | 0.92594 |  |
| > 24 °C | -0.00002 | (0.00002) | -0.85231 | 0.39455 |  | -0.00004 | (0.00002) | -2.24787 | 0.02513 | ** | -0.00007 | (0.00002) | -2.61271 | 0.00932 | *** | -0.00022 | (0.00006) | -3.61223 | 0.00034 | *** |
| Dementia#> 24 °C | -0.00022 | (0.00042) | -0.52203 | 0.60194 |  | 0.00022 | (0.00021) | 1.05720 | 0.29106 |  | 0.00012 | (0.00014) | 0.82977 | 0.40717 |  | 0.00107 | (0.00013) | 8.12975 | 0.00000 | *** |
| CCI without dementia | 0.00687 | (0.00020) | 33.62393 | 0.00000 | *** | 0.00990*** | (0.00016) | 61.88378 | 0.00000 |  | 0.01300 | (0.00016) | 82.85318 | 0.00000 | *** | 0.02076 | (0.00028) | 73.88345 | 0.00000 | *** |
| Wind | 0.00015 | (0.00016) | 0.96243 | 0.33642 |  | 0.00018 | (0.00017) | 1.07125 | 0.28471 |  | -0.00025 | (0.00025) | -0.99925 | 0.31828 |  | -0.00028 | (0.00054) | -0.50716 | 0.61232 |  |
| Rain | 0.00017 | (0.00007) | 2.28436 | 0.02288 | ** | 0.00006 | (0.00008) | 0.82403 | 0.41041 |  | -0.00000 | (0.00011) | -0.03318 | 0.97354 |  | -0.00045 | (0.00022) | -2.01502 | 0.04457 | ** |
| Solar radiation | 0.00000 | (0.00001) | 0.25913 | 0.79567 |  | 0.00001 | (0.00001) | 0.91777 | 0.35930 |  | 0.00000 | (0.00001) | 0.18072 | 0.85668 |  | 0.00006 | (0.00002) | 3.08456 | 0.00218 | *** |
| Relative Humidity | -0.00004 | (0.00003) | -1.59423 | 0.11168 |  | -0.00000 | (0.00003) | -0.17671 | 0.85982 |  | -0.00002 | (0.00003) | -0.49446 | 0.62125 |  | 0.00015 | (0.00007) | 2.27139 | 0.02366 | ** |
| PM2.5 | 0.00004 | (0.00003) | 1.16093 | 0.24636 |  | 0.00004 | (0.00003) | 1.12710 | 0.26038 |  | 0.00003 | (0.00004) | 0.73856 | 0.46061 |  | 0.00010 | (0.00008) | 1.14462 | 0.25305 |  |
| Constant | -0.00432 | (0.00273) | -1.58416 | 0.11395 |  | -0.01807*** | (0.00272) | -6.65051 | 0.00000 |  | -0.02764 | (0.00346) | -7.97723 | 0.00000 | *** | -0.07491 | (0.00752) | -9.96560 | 0.00000 | *** |
|  |  |  |  |  |  |  |  |  |  |  |  |  |  |  |  |  |  |  |  |  |
| Observations | 1,462,481 |  |  |  |  | 3,333,181 |  |  |  |  | 3,692,773 |  |  |  |  | 2,503,840 |  |  |  |  |
| R-squared | 0.12795 |  |  |  |  | 0.12205 |  |  |  |  | 0.12003 |  |  |  |  | 0.11555 |  |  |  |  |
| Adj. R-Squared. | 0.0855 |  |  |  |  | 0.0845 |  |  |  |  | 0.0823 |  |  |  |  | 0.0745 |  |  |  |  |
| F-stat | 46.06 |  |  |  |  | 172.5 |  |  |  |  | 362.1 |  |  |  |  | 470.9 |  |  |  |  |
| F-test | 0 |  |  |  |  | 0 |  |  |  |  | 0 |  |  |  |  | 0 |  |  |  |  |

Note: we present results for running an analysis as in Equation (2) separately by age categories:50-59;60-69;70-79;80+. Standard errors clustered at the Kreise level. Abbreviations: CCI: Charlson Comorbidity Index; Coeff: coefficient, F-Stat.: F-Statistics; Sign.: Significance. *** p<0.01, ** p<0.05, * p<0.1.

Table S5. Temperature, hospitalization separately by age and interaction with dementia

|  | Age 50-59 | |  |  |  |  | Age 60-69 |  |  |  |  | Age 70-79 |  |  |  |  | Age 80+ |  |  |  |  |
| --- | --- | --- | --- | --- | --- | --- | --- | --- | --- | --- | --- | --- | --- | --- | --- | --- | --- | --- | --- | --- | --- |
| Variables | | Coeff. | Stand. Error | T-Stat. | P-value | Sign. | Coeff. | Stand. Error | T-Stat. | P-value | Sign. | Coeff. | Stand. Error | T-Stat. | P-value | Sign. | Coeff. | Stand. Error | T-Stat. | P-value | Sign. |
| < -6 °C | | 0.00022 | (0.00009) | 2.55110 | 0.01111 | ** | -0.00001 | (0.00007) | -0.07813 | 0.93776 |  | 0.00003 | (0.00008) | 0.33592 | 0.73711 |  | 0.00026 | (0.00012) | 2.15612 | 0.03167 | ** |
| Dementia | | 0.06436 | (0.02477) | 2.59848 | 0.00971 | *** | 0.06627 | (0.01012) | 6.55134 | 0.00000 | *** | 0.07103 | (0.00587) | 12.10846 | 0.00000 | *** | 0.08560 | (0.00463) | 18.48920 | 0.00000 | *** |
| Dementia# < -6 °C | | -0.00168 | (0.00105) | -1.59917 | 0.11057 |  | -0.00061 | (0.00058) | -1.04390 | 0.29716 |  | -0.00004 | (0.00032) | -0.13498 | 0.89269 |  | 0.00021 | (0.00024) | 0.85253 | 0.39443 |  |
| -6 °C to -3 °C | | -0.00017 | (0.00007) | -2.30895 | 0.02146 | ** | 0.00007 | (0.00006) | 1.05523 | 0.29196 |  | 0.00012 | (0.00007) | 1.74780 | 0.08127 | * | 0.00024 | (0.00010) | 2.48037 | 0.01354 | ** |
| Dementia# -6 °C to -3 °C | | -0.00111 | (0.00101) | -1.09260 | 0.27523 |  | 0.00069 | (0.00049) | 1.40407 | 0.16108 |  | -0.00009 | (0.00025) | -0.33733 | 0.73605 |  | -0.00029 | (0.00020) | -1.43371 | 0.15244 |  |
| -3 °C to 0 °C | | -0.00004 | (0.00006) | -0.59163 | 0.55443 |  | -0.00001 | (0.00005) | -0.24163 | 0.80919 |  | 0.00007 | (0.00006) | 1.31416 | 0.18955 |  | 0.00011 | (0.00008) | 1.36256 | 0.17379 |  |
| Dementia#-3 °C to 0 °C | | -0.00021 | (0.00081) | -0.25824 | 0.79635 |  | -0.00066 | (0.00037) | -1.79330 | 0.07368 | * | 0.00049 | (0.00021) | 2.33254 | 0.02017 | ** | 0.00065 | (0.00015) | 4.31666 | 0.00002 | *** |
| 0 °C to 3 °C | | -0.00007 | (0.00006) | -1.20205 | 0.23006 |  | -0.00008 | (0.00004) | -1.93761 | 0.05338 | * | 0.00001 | (0.00005) | 0.27859 | 0.78070 |  | 0.00024 | (0.00007) | 3.42189 | 0.00069 | *** |
| Dementia#0 °C to 3 °C | | -0.00053 | (0.00074) | -0.71337 | 0.47604 |  | 0.00029 | (0.00029) | 1.00642 | 0.31482 |  | -0.00019 | (0.00017) | -1.08337 | 0.27930 |  | -0.00002 | (0.00012) | -0.13463 | 0.89297 |  |
| 3 °C to 6 °C | | -0.00008 | (0.00005) | -1.64092 | 0.10160 |  | 0.00005 | (0.00004) | 1.33379 | 0.18304 |  | 0.00006 | (0.00004) | 1.51427 | 0.13075 |  | 0.00027 | (0.00006) | 4.36065 | 0.00002 | *** |
| Dementia#3 °C to 6 °C | | -0.00003 | (0.00065) | -0.04397 | 0.96495 |  | -0.00018 | (0.00024) | -0.72570 | 0.46845 |  | -0.00011 | (0.00016) | -0.69131 | 0.48977 |  | -0.00010 | (0.00010) | -0.98647 | 0.32450 |  |
| 6 °C to 9 °C | | -0.00008 | (0.00005) | -1.68471 | 0.09283 | * | 0.00002 | (0.00004) | 0.47744 | 0.63331 |  | 0.00009 | (0.00004) | 2.11459 | 0.03508 | ** | 0.00024 | (0.00006) | 3.87217 | 0.00013 | *** |
| Dementia#6 °C to 9 °C | | -0.00132 | (0.00074) | -1.80113 | 0.07244 | * | -0.00026 | (0.00028) | -0.90262 | 0.36727 |  | -0.00018 | (0.00017) | -1.03882 | 0.29952 |  | -0.00014 | (0.00012) | -1.14189 | 0.25418 |  |
| 16 °C to 18 °C | | -0.00005 | (0.00006) | -0.95105 | 0.34215 |  | 0.00000 | (0.00003) | 0.04574 | 0.96354 |  | -0.00000 | (0.00004) | -0.10963 | 0.91275 |  | 0.00004 | (0.00007) | 0.59064 | 0.55510 |  |
| Dementia#16 °C to 18 °C | | -0.00192 | (0.00073) | -2.63977 | 0.00862 | *** | 0.00003 | (0.00032) | 0.08534 | 0.93204 |  | 0.00002 | (0.00018) | 0.08780 | 0.93008 |  | -0.00004 | (0.00014) | -0.31186 | 0.75531 |  |
| 18 °C to 21 °C | | 0.00007 | (0.00006) | 1.23959 | 0.21585 |  | 0.00005 | (0.00004) | 1.30054 | 0.19417 |  | 0.00001 | (0.00005) | 0.23323 | 0.81570 |  | 0.00006 | (0.00007) | 0.87979 | 0.37950 |  |
| Dementia#18 °C to 21 °C | | -0.00011 | (0.00065) | -0.16730 | 0.86722 |  | -0.00034 | (0.00029) | -1.16124 | 0.24624 |  | -0.00035 | (0.00018) | -1.90091 | 0.05803 | * | -0.00015 | (0.00013) | -1.16879 | 0.24318 |  |
| 21 °C to 24 °C | | -0.00006 | (0.00007) | -0.86798 | 0.38592 |  | 0.00004 | (0.00005) | 0.81450 | 0.41584 |  | -0.00012 | (0.00007) | -1.74048 | 0.08255 | * | -0.00028 | (0.00010) | -2.83068 | 0.00488 | *** |
| Dementia#21 °C to 24 °C | | 0.00258 | (0.00107) | 2.40558 | 0.01660 | ** | -0.00004 | (0.00047) | -0.08296 | 0.93393 |  | 0.00044 | (0.00026) | 1.69091 | 0.09164 | * | 0.00003 | (0.00022) | 0.15437 | 0.87740 |  |
| > 24 °C | | 0.00004 | (0.00011) | 0.33436 | 0.73828 |  | 0.00003 | (0.00008) | 0.32386 | 0.74621 |  | -0.00015 | (0.00008) | -1.88694 | 0.05989 | * | 0.00004 | (0.00012) | 0.29498 | 0.76816 |  |
| Dementia#> 24 °C | | -0.00280 | (0.00162) | -1.73196 | 0.08405 | * | -0.00017 | (0.00062) | -0.27480 | 0.78361 |  | -0.00018 | (0.00031) | -0.58832 | 0.55665 |  | -0.00004 | (0.00028) | -0.13984 | 0.88885 |  |
| CCI without dementia | | 0.03355 | (0.00067) | 50.38192 | 0.00000 | *** | 0.04038 | (0.00047) | 86.54759 | 0.00000 | *** | 0.04330 | (0.00039) | 110.38233 | 0.00000 | *** | 0.04612 | (0.00043) | 106.67324 | 0.00000 | *** |
| Wind | | 0.00066 | (0.00089) | 0.74270 | 0.45810 |  | -0.00019 | (0.00063) | -0.29866 | 0.76536 |  | -0.00015 | (0.00075) | -0.19477 | 0.84567 |  | -0.00030 | (0.00097) | -0.30755 | 0.75858 |  |
| Rain | | -0.00008 | (0.00040) | -0.19849 | 0.84277 |  | 0.00043 | (0.00030) | 1.43429 | 0.15227 |  | 0.00072 | (0.00030) | 2.36973 | 0.01828 | ** | 0.00082* | (0.00045) | 1.82398 | 0.06890 |  |
| Solar radiation | | -0.00011 | (0.00004) | -3.16001 | 0.00170 | *** | -0.00002 | (0.00003) | -0.58031 | 0.56203 |  | 0.00002 | (0.00003) | 0.86170 | 0.38937 |  | 0.00005 | (0.00004) | 1.30088 | 0.19405 |  |
| Relative Humidity | | -0.00018 | (0.00012) | -1.46345 | 0.14413 |  | -0.00001 | (0.00011) | -0.12960 | 0.89695 |  | -0.00018 | (0.00010) | -1.80260 | 0.07221 | * | 0.00003 | (0.00014) | 0.24370 | 0.80759 |  |
| PM2.5 | | -0.00004 | (0.00016) | -0.22131 | 0.82497 |  | 0.00004 | (0.00012) | 0.32392 | 0.74617 |  | -0.00011 | (0.00014) | -0.80981 | 0.41853 |  | 0.00011 | (0.00017) | 0.65868 | 0.51048 |  |
| Constant | | 0.04192 | (0.01295) | 3.23827 | 0.00130 | *** | -0.00955 | (0.01067) | -0.89428 | 0.37171 |  | -0.01224 | (0.01023) | -1.19635 | 0.23227 |  | -0.06666 | (0.01510) | -4.41460 | 0.00001 | *** |
| Observations | | 1,462,481 |  |  |  |  | 3,333,181 |  |  |  |  | 3,692,773 |  |  |  |  | 2,503,840 |  |  |  |  |
| R-squared | | 0.15726 |  |  |  |  | 0.16881 |  |  |  |  | 0.16758 |  |  |  |  | 0.15677 |  |  |  |  |
| Adj. R-Squared. | | 0.116 |  |  |  |  | 0.133 |  |  |  |  | 0.132 |  |  |  |  | 0.118 |  |  |  |  |
| F-stat | | 112.2 |  |  |  |  | 359 |  |  |  |  | 573.5 |  |  |  |  | 553.9 |  |  |  |  |
| F-test | | 0 |  |  |  |  | 0 |  |  |  |  | 0 |  |  |  |  | 0 |  |  |  |  |

Note: we present results for running an analysis as in Equation (2) separately by age categories:50-59;60-69;70-79;80+. Standard errors clustered at the Kreise level. Abbreviations: CCI: Charlson Comorbidity Index; Coeff: coefficient, F-Stat.: F-Statistics; Sign.: Significance. *** p<0.01, ** p<0.05, * p<0.1.

Table S6. Temperature, mortality, hospitalization and lagged effects

|  | Mortality |  |  | |  | |  | | Hospitalization | |  | |  | |  | | |  |  |
| --- | --- | --- | --- | --- | --- | --- | --- | --- | --- | --- | --- | --- | --- | --- | --- | --- | --- | --- | --- |
| Variables | Coeff. | Standard Error | | T-Stat. | | P-value | | Sign. | | Coeff. | | Standard Error | | T-Stat. | | P-value | Sign. | | |
|  |  |  |  | |  | |  | |  | |  | |  | |  | | |  |  |
| < -6 °C | 0.00013 | (0.00002) | 7.34897 | | 0.00000 | | *** | | 0.00018 | | (0.00005) | | 3.84603 | | 0.00014 | | | *** |  |
| < -6 °C lagged | 0.00001 | (0.00002) | 0.56994 | | 0.56904 | |  | | 0.00004 | | (0.00004) | | 0.84944 | | 0.39615 | | |  |  |
| -6 °C to -3 °C | 0.00014 | (0.00002) | 9.00771 | | 0.00000 | | *** | | 0.00016 | | (0.00004) | | 3.71813 | | 0.00023 | | | *** |  |
| -6 °C to -3 °C lagged | 0.00000 | (0.00001) | 0.27783 | | 0.78128 | |  | | 0.00010 | | (0.00004) | | 2.81831 | | 0.00507 | | | *** |  |
| -3 °C to 0 °C | -0.00002 | (0.00001) | -1.43783 | | 0.15127 | |  | | 0.00009 | | (0.00003) | | 2.75237 | | 0.00619 | | | *** |  |
| -3 °C to 0 °C lagged | -0.00007 | (0.00001) | -6.32303 | | 0.00000 | | *** | | 0.00004 | | (0.00003) | | 1.43772 | | 0.15130 | | |  |  |
| 0 °C to 3 °C | 0.00005 | (0.00001) | 4.78809 | | 0.00000 | | *** | | 0.00005 | | (0.00003) | | 1.97794 | | 0.04862 | | | ** |  |
| 0 °C to 3 °C lagged | -0.00004 | (0.00001) | -4.29201 | | 0.00002 | | *** | | 0.00004 | | (0.00002) | | 1.44620 | | 0.14891 | | |  |  |
| 3 °C to 6 °C | 0.00006 | (0.00001) | 6.35916 | | 0.00000 | | *** | | 0.00011 | | (0.00002) | | 4.47547 | | 0.00001 | | | *** |  |
| 3 °C to 6 °C lagged | -0.00008 | (0.00001) | -8.75853 | | 0.00000 | | *** | | 0.00003 | | (0.00002) | | 1.57709 | | 0.11557 | | |  |  |
| 6 °C to 9 °C | 0.00004 | (0.00001) | 4.00875 | | 0.00007 | | *** | | 0.00011 | | (0.00003) | | 3.97211 | | 0.00008 | | | *** |  |
| 6 °C to 9 °C lagged | -0.00001 | (0.00001) | -0.66529 | | 0.50625 | |  | | 0.00009 | | (0.00002) | | 3.63305 | | 0.00032 | | | *** |  |
| 16 °C to 18 °C | 0.00001 | (0.00001) | 0.95383 | | 0.34075 | |  | | 0.00001 | | (0.00002) | | 0.34636 | | 0.72926 | | |  |  |
| 16 °C to 18 °C lagged | -0.00001 | (0.00001) | -1.26333 | | 0.20721 | |  | | 0.00001 | | (0.00002) | | 0.49111 | | 0.62362 | | |  |  |
| 18 °C to 21 °C | -0.00007 | (0.00001) | -6.67072 | | 0.00000 | | *** | | 0.00001 | | (0.00003) | | 0.28322 | | 0.77716 | | |  |  |
| 18 °C to 21 °C lagged | -0.00004 | (0.00001) | -4.42851 | | 0.00001 | | *** | | -0.00001 | | (0.00003) | | -0.29811 | | 0.76578 | | |  |  |
| 21 °C to 24 °C | -0.00003 | (0.00001) | -2.16749 | | 0.03079 | | ** | | -0.00011 | | (0.00004) | | -2.93103 | | 0.00357 | | | *** |  |
| 21 °C to 24 °C lagged | 0.00003 | (0.00001) | 2.24874 | | 0.02507 | | ** | | -0.00002 | | (0.00004) | | -0.59368 | | 0.55306 | | |  |  |
| > 24 °C | -0.00006 | (0.00002) | -2.61870 | | 0.00916 | | *** | | -0.00011 | | (0.00006) | | -2.00942 | | 0.04517 | | | ** |  |
| > 24 °C lagged | -0.00012 | (0.00002) | -5.50640 | | 0.00000 | | *** | | -0.00008 | | (0.00005) | | -1.39866 | | 0.16269 | | |  |  |
| CCI | 0.01196 | (0.00011) | 109.23133 | | 0.00000 | | *** | | 0.03408 | | (0.00024) | | 139.41160 | | 0.00000 | | | *** |  |
| Wind | 0.00006 | (0.00019) | 0.32764 | | 0.74336 | |  | | 0.00021 | | (0.00049) | | 0.43575 | | 0.66325 | | |  |  |
| Rain | 0.00003 | (0.00007) | 0.46038 | | 0.64549 | |  | | 0.00049 | | (0.00018) | | 2.78071 | | 0.00568 | | | *** |  |
| Solar radiation | 0.00003 | (0.00001) | 4.14842 | | 0.00004 | | *** | | 0.00001 | | (0.00002) | | 0.48556 | | 0.62755 | | |  |  |
| Relative Humidity | -0.00003 | (0.00002) | -1.24417 | | 0.21417 | |  | | -0.00013 | | (0.00007) | | -1.94165 | | 0.05288 | | | * |  |
| PM2.5 | 0.00011 | (0.00003) | 3.40970 | | 0.00072 | | *** | | -0.00008 | | (0.00011) | | -0.76512 | | 0.44465 | | |  |  |
| Constant | -0.02297 | (0.00273) | -8.42366 | | 0.00000 | | *** | | 0.00465 | | (0.00702) | | 0.66277 | | 0.50786 | | |  |  |
|  |  |  |  | |  | |  | |  | |  | |  | |  | | |  |  |
| Observations | 11,001,659 |  |  | |  | |  | | 11,001,659 | |  | |  | |  | | |  |  |
| R-squared | 0.08527 |  |  | |  | |  | | 0.13571 | |  | |  | |  | | |  |  |
| Adj. R-Squared. | 0.0645 |  |  | |  | |  | | 0.116 | |  | |  | |  | | |  |  |
| F-stat | 656.7 |  |  | |  | |  | | 923.7 | |  | |  | |  | | |  |  |
| F-test | 0 |  |  | |  | |  | | 0 | |  | |  | |  | | |  |  |

Note: we present results for running an analysis as in Equation (1) with lagged values for temperature exposures. Standard errors clustered at the Kreise level. Abbreviations: CCI: Charlson Comorbidity Index; Coeff: coefficient, F-Stat.: F-Statistics; Sign.: Significance. *** p<0.01, ** p<0.05, * p<0.1.

Table S7. Temperature and emergency hospitalization

| Variables | Coeff. | Standard Error | T-Stat. | P-value | Sign. |
| --- | --- | --- | --- | --- | --- |
|  |  |  |  |  |  |
| < -6 °C | 0.00009 | (0.00004) | 2.26685 | 0.02393 | ** |
| -6 °C to -3 °C | 0.00009 | (0.00004) | 2.29091 | 0.02249 | ** |
| -3 °C to 0 °C | 0.00004 | (0.00002) | 1.52656 | 0.12766 |  |
| 0 °C to 3 °C | 0.00003 | (0.00002) | 1.16989 | 0.24274 |  |
| 3 °C to 6 °C | 0.00006 | (0.00002) | 3.02440 | 0.00265 | *** |
| 6 °C to 9 °C | 0.00003 | (0.00002) | 1.28087 | 0.20098 |  |
| 16 °C to 18 °C | -0.00002 | (0.00002) | -1.27169 | 0.20422 |  |
| 18 °C to 21 °C | -0.00000 | (0.00002) | -0.21420 | 0.83050 |  |
| 21 °C to 24 °C | -0.00002 | (0.00003) | -0.84836 | 0.39674 |  |
| > 24 °C | -0.00018 | (0.00004) | -3.97721 | 0.00008 | *** |
| CCI | 0.01818 | (0.00039) | 46.80318 | 0.00000 | *** |
| Wind | -0.00024 | (0.00062) | -0.38886 | 0.69759 |  |
| Rain | 0.00000 | (0.00017) | 0.02016 | 0.98393 |  |
| Solar radiation | 0.00000 | (0.00002) | 0.10811 | 0.91396 |  |
| Relative Humidity | -0.00003 | (0.00008) | -0.38580 | 0.69985 |  |
| PM2.5 | -0.00026 | (0.00013) | -1.97291 | 0.04920 | ** |
| Constant | -0.00260 | (0.00777) | -0.33491 | 0.73787 |  |
|  |  |  |  |  |  |
| Observations | 11,001,659 |  |  |  |  |
| R-squared | 0.10067 |  |  |  |  |
| Adj. R-Squared. | 0.0803 |  |  |  |  |
| F-stat | 292.2 |  |  |  |  |
| F-test | 0 |  |  |  |  |

Note: we present results for running an analysis as in Equation (1) with emergency hospitalizations as the outcome. Standard errors clustered at the Kreise level. Standard errors in parentheses. Abbreviations: CCI: Charlson Comorbidity Index; Coeff: coefficient, F-Stat.: F-Statistics; Sign.: Significance. *** p<0.01, ** p<0.05, * p<0.1.

Table S8. Relative temperature, mortality and hospitalization

|  | Mortality |  |  |  |  | Hospitalization |  |  |  |  |
| --- | --- | --- | --- | --- | --- | --- | --- | --- | --- | --- |
| Variables | Coeff. | Standard Error | T-Stat. | P-value | Sign. | Coeff. | Standard Error | T-Stat. | P-value | Sign. |
|  |  |  |  |  |  |  |  |  |  |  |
| < 5th percentile | 0.00006 | (0.00001) | 7.72572 | 0.00000 | *** | 0.00005 | (0.00002) | 2.31686 | 0.02102 | ** |
| 5th to 10th percentile | -0.00011 | (0.00001) | -9.03876 | 0.00000 | *** | -0.00004 | (0.00003) | -1.28705 | 0.19882 |  |
| 10th to 15th percentile | 0.00005 | (0.00002) | 2.97058 | 0.00315 | *** | 0.00009 | (0.00004) | 2.23725 | 0.02582 | ** |
| 15th to 20th percentile | 0.00008 | (0.00002) | 4.81580 | 0.00000 | *** | 0.00006 | (0.00004) | 1.60357 | 0.10960 |  |
| 75th to 80th percentile | -0.00001 | (0.00001) | -0.45226 | 0.65133 |  | 0.00007 | (0.00004) | 1.82470 | 0.06879 | * |
| 80 to 85th percentile | -0.00005 | (0.00002) | -3.02506 | 0.00265 | *** | 0.00003 | (0.00004) | 0.71060 | 0.47775 |  |
| 85th to 90th percentile | -0.00003 | (0.00001) | -2.34379 | 0.01958 | ** | -0.00009 | (0.00004) | -2.57443 | 0.01040 | ** |
| > 95th percentile | -0.00002 | (0.00001) | -1.40241 | 0.16157 |  | -0.00004 | (0.00003) | -1.31187 | 0.19032 |  |
| CCI | 0.01196 | (0.00011) | 109.18452 | 0.00000 | *** | 0.03408 | (0.00024) | 139.34054 | 0.00000 | *** |
| Wind | -0.00011 | (0.00018) | -0.60078 | 0.54833 |  | 0.00004 | (0.00048) | 0.07313 | 0.94174 |  |
| Rain | -0.00009 | (0.00007) | -1.40287 | 0.16143 |  | 0.00028 | (0.00017) | 1.64075 | 0.10164 |  |
| Solar radiation | 0.00001 | (0.00001) | 1.93472 | 0.05373 | * | -0.00000 | (0.00002) | -0.06688 | 0.94671 |  |
| Relative Humidity | 0.00002 | (0.00002) | 0.89615 | 0.37071 |  | -0.00007 | (0.00007) | -1.06489 | 0.28757 |  |
| PM2.5 | 0.00010 | (0.00003) | 3.20177 | 0.00148 | *** | -0.00008 | (0.00011) | -0.70734 | 0.47977 |  |
| Constant | -0.02460 | (0.00243) | -10.11185 | 0.00000 | *** | 0.00739 | (0.00658) | 1.12264 | 0.26227 |  |
|  |  |  |  |  |  |  |  |  |  |  |
| Observations | 11,001,659 |  |  |  |  | 11,001,659 |  |  |  |  |
| R-squared | 0.08524 |  |  |  |  | 0.13571 |  |  |  |  |
| Adj. R-Squared. | 0.0645 |  |  |  |  | 0.116 |  |  |  |  |
| F-stat | 967.8 |  |  |  |  | 1441 |  |  |  |  |
| F-test | 0 |  |  |  |  | 0 |  |  |  |  |

Note: we present results for running an analysis as in Equation (1) relative temperature bins for temperature exposures. Standard errors clustered at the Kreise level. Abbreviations: CCI: Charlson Comorbidity Index; Coeff: coefficient, F-Stat.: F-Statistics; Sign.: Significance. *** p<0.01, ** p<0.05, * p<0.1.

Table S9. Alternative modeling specifications mortality

|  | Model (1) |  |  |  |  | Model (2) | (6) | (7) | (8) |  | Model (3) |  |  |  |  |  |
| --- | --- | --- | --- | --- | --- | --- | --- | --- | --- | --- | --- | --- | --- | --- | --- | --- |
| Variables | Coeff. | Standard Error | T-Stat. | P-value | Sign. | Coeff. | Standard Error | T-Stat. | P-value | Sign. | Coeff. | Standard Error | T-Stat. | P-value | Sign. |  |
|  |  |  |  |  |  |  |  |  |  |  |  |  |  |  |  |  |
| < -6 °C | 0.00011 | (0.00002) | 6.41361 | 0.00000 | *** | 0.00010 | (0.00002) | 6.06076 | 0.00000 | *** | 0.00011 | (0.00002) | 5.80519 | 0.00000 | *** |  |
| -6 °C to -3 °C | 0.00016 | (0.00002) | 10.26366 | 0.00000 | *** | 0.00014 | (0.00002) | 9.58893 | 0.00000 | *** | 0.00016 | (0.00002) | 10.21402 | 0.00000 | *** |  |
| -3 °C to 0 °C | -0.00001 | (0.00001) | -0.69639 | 0.48659 |  | -0.00001 | (0.00001) | -0.43899 | 0.66090 |  | -0.00000 | (0.00001) | -0.30715 | 0.75889 |  |  |
| 0 °C to 3 °C | 0.00007 | (0.00001) | 7.32869 | 0.00000 | *** | 0.00006 | (0.00001) | 6.52139 | 0.00000 | *** | 0.00008 | (0.00001) | 7.38390 | 0.00000 | *** |  |
| 3 °C to 6 °C | 0.00008 | (0.00001) | 8.54607 | 0.00000 | *** | 0.00008 | (0.00001) | 8.58087 | 0.00000 | *** | 0.00008 | (0.00001) | 8.36965 | 0.00000 | *** |  |
| 6 °C to 9 °C | 0.00007 | (0.00001) | 6.50009 | 0.00000 | *** | 0.00006 | (0.00001) | 5.60680 | 0.00000 | *** | 0.00007 | (0.00001) | 6.88780 | 0.00000 | *** |  |
| 16 °C to 18 °C | 0.00002 | (0.00001) | 1.60055 | 0.11027 |  | 0.00001 | (0.00001) | 1.26708 | 0.20586 |  | 0.00001 | (0.00001) | 0.66905 | 0.50385 |  |  |
| 18 °C to 21 °C | -0.00005 | (0.00001) | -4.36545 | 0.00002 | *** | -0.00004 | (0.00001) | -4.05548 | 0.00006 | *** | -0.00006 | (0.00001) | -6.09288 | 0.00000 | *** |  |
| 21 °C to 24 °C | 0.00000 | (0.00001) | 0.02195 | 0.98250 |  | 0.00000 | (0.00001) | 0.08014 | 0.93616 |  | -0.00002 | (0.00002) | -1.42428 | 0.15515 |  |  |
| > 24 °C | -0.00004 | (0.00002) | -2.22358 | 0.02674 | ** | -0.00004 | (0.00002) | -1.82143 | 0.06929 | * | -0.00006 | (0.00002) | -3.36213 | 0.00085 | *** |  |
| CCI | 0.01196 | (0.00011) | 109.23972 | 0.00000 | *** | 0.01107 | (0.00011) | 101.66402 | 0.00000 | *** | 0.01196 | (0.00011) | 109.35103 | 0.00000 | *** |  |
| Wind | 0.00004 | (0.00019) | 0.23121 | 0.81727 |  | -0.00005 | (0.00019) | -0.28339 | 0.77703 |  | 0.00013 | (0.00022) | 0.58059 | 0.56185 |  |  |
| Rain | 0.00005 | (0.00007) | 0.74096 | 0.45915 |  | -0.00002 | (0.00007) | -0.24535 | 0.80631 |  | 0.00001 | (0.00008) | 0.18028 | 0.85703 |  |  |
| Solar radiation | 0.00002 | (0.00001) | 3.16072 | 0.00169 | *** | 0.00002 | (0.00001) | 3.96855 | 0.00009 | *** | 0.00003 | (0.00001) | 4.50236 | 0.00001 | *** |  |
| Relative Humidity | -0.00002 | (0.00002) | -0.66787 | 0.50460 |  | 0.00002 | (0.00002) | 0.84292 | 0.39978 |  | -0.00000 | (0.00003) | -0.04560 | 0.96365 |  |  |
| PM2.5 | 0.00012 | (0.00003) | 3.84724 | 0.00014 | *** | 0.00001 | (0.00003) | 0.29558 | 0.76770 |  | 0.00013 | (0.00003) | 3.71478 | 0.00023 | *** |  |
| Constant | -0.02671 | (0.00253) | -10.53897 | 0.00000 | *** | -0.02526 | (0.00252) | -10.01836 | 0.00000 | *** | -0.02956 | (0.00335) | -8.82215 | 0.00000 | *** |  |
|  |  |  |  |  |  |  |  |  |  |  |  |  |  |  |  |  |
| Observations | 11,001,659 |  |  |  |  | 11,001,659 |  |  |  |  | 11,001,659 |  |  |  |  |  |
| R-squared | 0.08525 |  |  |  |  | 0.09256 |  |  |  |  | 0.08547 |  |  |  |  |  |
| Adj. R-Squared. | 0.0645 |  |  |  |  | 0.0720 |  |  |  |  | 0.0646 |  |  |  |  |  |
| F-stat | 992.3 |  |  |  |  | 833.6 |  |  |  |  | 963.7 |  |  |  |  |  |
| F-test | 0 |  |  |  |  | 0 |  |  |  |  |  |  |  |  |  |  |
| Individual FE | YES |  |  |  |  | YES |  |  |  |  | YES |  |  |  |  |  |
| Month FE | YES |  |  |  |  | YES |  |  |  |  | NO |  |  |  |  |  |
| Year FE | YES |  |  |  |  | YES |  |  |  |  | YES |  |  |  |  |  |
| Kreise by month FE | NO |  |  |  |  | NO |  |  |  |  | YES |  |  |  |  |  |
| Age FE | NO |  |  |  |  | YES |  |  |  |  | NO |  |  |  |  |  |

Note: we present results for running an analysis as in Equation (1), but with different model specifications. Standard errors clustered at the Kreise level. Standard errors in parentheses. Abbreviations: CCI: Charlson Comorbidity Index; Coeff: coefficient, F-Stat.: F-Statistics; Sign.: Significance. *** p<0.01, ** p<0.05, * p<0.1.

Table S10. Alternative modeling specifications hospitalization

|  | Model (1) |  |  |  |  | Model (2) |  |  |  |  | Model (3) |  |  |  |  |
| --- | --- | --- | --- | --- | --- | --- | --- | --- | --- | --- | --- | --- | --- | --- | --- |
| Variables | Coeff. | Standard Error | T-Stat. | P-value | Sign. | Coeff. | Standard Error | T-Stat. | P-value | Sign. | Coeff. | Standard Error | T-Stat. | P-value | Sign. |
|  |  |  |  |  |  |  |  |  |  |  |  |  |  |  |  |
| < -6 °C | 0.00017 | (0.00004) | 3.75217 | 0.00020 | *** | 0.00016 | (0.00004) | 3.69816 | 0.00025 | *** | 0.00018 | (0.00005) | 3.70380 | 0.00024 | *** |
| -6 °C to -3 °C | 0.00016 | (0.00004) | 3.69814 | 0.00025 | *** | 0.00015 | (0.00004) | 3.65382 | 0.00029 | *** | 0.00017 | (0.00004) | 3.90102 | 0.00011 | *** |
| -3 °C to 0 °C | 0.00010 | (0.00003) | 3.12253 | 0.00192 | *** | 0.00010 | (0.00003) | 3.16245 | 0.00168 | *** | 0.00011 | (0.00003) | 3.36772 | 0.00083 | *** |
| 0 °C to 3 °C | 0.00006 | (0.00003) | 2.20877 | 0.02776 | ** | 0.00006 | (0.00003) | 2.14431 | 0.03261 | ** | 0.00008 | (0.00003) | 2.78920 | 0.00554 | *** |
| 3 °C to 6 °C | 0.00011 | (0.00002) | 4.61075 | 0.00001 | *** | 0.00011 | (0.00002) | 4.61024 | 0.00001 | *** | 0.00012 | (0.00002) | 5.09235 | 0.00000 | *** |
| 6 °C to 9 °C | 0.00010 | (0.00003) | 4.01959 | 0.00007 | *** | 0.00010 | (0.00003) | 3.96754 | 0.00009 | *** | 0.00011 | (0.00003) | 4.20556 | 0.00003 | *** |
| 16 °C to 18 °C | 0.00001 | (0.00002) | 0.46136 | 0.64479 |  | 0.00001 | (0.00002) | 0.43233 | 0.66573 |  | 0.00000 | (0.00002) | 0.14880 | 0.88179 |  |
| 18 °C to 21 °C | 0.00001 | (0.00003) | 0.19680 | 0.84409 |  | 0.00001 | (0.00003) | 0.22946 | 0.81863 |  | 0.00001 | (0.00003) | 0.27662 | 0.78222 |  |
| 21 °C to 24 °C | -0.00011 | (0.00004) | -2.90496 | 0.00388 | *** | -0.00011 | (0.00004) | -2.89642 | 0.00398 | *** | -0.00010 | (0.00004) | -2.51339 | 0.01235 | ** |
| > 24 °C | -0.00011 | (0.00005) | -2.08280 | 0.03791 | ** | -0.00011 | (0.00005) | -2.05996 | 0.04005 | ** | -0.00011 | (0.00006) | -1.83692 | 0.06696 | * |
| CCI | 0.03408 | (0.00024) | 139.39563 | 0.00000 | *** | 0.03385 | (0.00025) | 133.25112 | 0.00000 | *** | 0.03408 | (0.00024) | 139.27235 | 0.00000 | *** |
| Wind | 0.00013 | (0.00049) | 0.25680 | 0.79746 |  | 0.00011 | (0.00049) | 0.21934 | 0.82650 |  | 0.00019 | (0.00062) | 0.30344 | 0.76171 |  |
| Rain | 0.00056 | (0.00018) | 3.17305 | 0.00163 | *** | 0.00054 | (0.00018) | 3.07197 | 0.00227 | *** | 0.00057 | (0.00020) | 2.78513 | 0.00561 | *** |
| Solar radiation | 0.00001 | (0.00002) | 0.77843 | 0.43677 |  | 0.00001 | (0.00002) | 0.86403 | 0.38809 |  | 0.00001 | (0.00002) | 0.82888 | 0.40767 |  |
| Relative Humidity | -0.00014 | (0.00007) | -2.02586 | 0.04344 | ** | -0.00013 | (0.00007) | -1.89563 | 0.05873 | * | -0.00013 | (0.00008) | -1.56643 | 0.11804 |  |
| PM2.5 | -0.00006 | (0.00011) | -0.50154 | 0.61627 |  | -0.00009 | (0.00011) | -0.77268 | 0.44017 |  | -0.00006 | (0.00013) | -0.43951 | 0.66053 |  |
| Constant | 0.00656 | (0.00682) | 0.96220 | 0.33653 |  | 0.00684 | (0.00681) | 1.00520 | 0.31541 |  | 0.00455 | (0.00806) | 0.56503 | 0.57237 |  |
|  |  |  |  |  |  |  |  |  |  |  |  |  |  |  |  |
| Observations | 11,001,659 |  |  |  |  | 11,001,659 |  |  |  |  | 11,001,659 |  |  |  |  |
| R-squared | 0.13571 |  |  |  |  | 0.13575 |  |  |  |  | 0.13590 |  |  |  |  |
| Adj. R-Squared. | 0.116 |  |  |  |  | 0.116 |  |  |  |  | 0.116 |  |  |  |  |
| F-stat | 1412 |  |  |  |  | 1293 |  |  |  |  | 1346 |  |  |  |  |
| F-test | 0 |  |  |  |  | 0 |  |  |  |  | 0 |  |  |  |  |
|  |  |  |  |  |  |  |  |  |  |  |  |  |  |  |  |
| Individual FE | YES |  |  |  |  | YES |  |  |  |  | YES |  |  |  |  |
| Month FE | YES |  |  |  |  | YES |  |  |  |  | NO |  |  |  |  |
| Year FE | YES |  |  |  |  | YES |  |  |  |  | YES |  |  |  |  |
| Kreise by month FE | NO |  |  |  |  | NO |  |  |  |  | YES |  |  |  |  |
| Age FE | NO |  |  |  |  | YES |  |  |  |  | NO |  |  |  |  |

Note: we present results for running an analysis as in Equation (1), but with different model specifications. Standard errors clustered at the Kreise level. Standard errors in parentheses. Abbreviations: CCI: Charlson Comorbidity Index; Coeff: coefficient, F-Stat.: F-Statistics; Sign.: Significance. *** p<0.01, ** p<0.05, * p<0.1.

**Figures**

Figure S1. Map of mean temperature in Germany 2004-2019


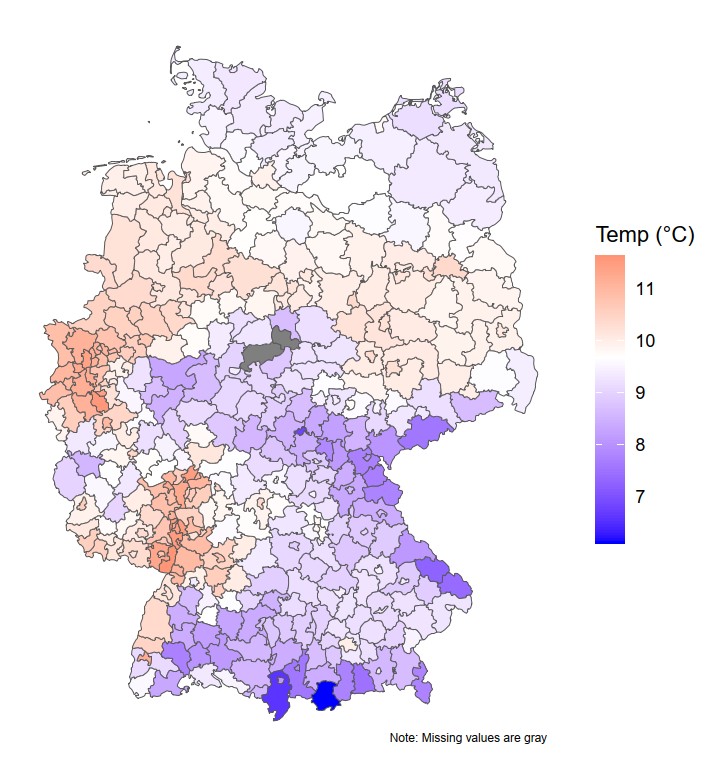


Note:in the figure we plot the average temperature in the German Kreise across our period of analysis 2004-2019. In gray are denoted the Kreise for which we lack AOK data. The average area of each Kreise is about 888 square kilometers, with the largest being 5,503 square kilometers, called Mecklenburgische Seenplatte and located in the North-East of the country, whereas the smallest is called Schweinfurt of 36 square kilometers and located in the central part of Germany. The values in the map show higher average temperatures in the western parts of the country and present colder areas in the South and South-East where mountains are located.
